# Macrophages directly kill bladder cancer cells through TNF signaling as an early response to BCG therapy

**DOI:** 10.1242/dmm.050693

**Published:** 2024-08-08

**Authors:** Mayra Fernanda Martínez-López, Cátia Rebelo de Almeida, Márcia Fontes, Raquel Valente Mendes, Stefan H. E. Kaufmann, Rita Fior

**Affiliations:** ^1^ Cancer Research Group (CRG), Faculty of Medicine, Universidad de las Américas, Quito 170124, Ecuador; ^2^ Champalimaud Research, Champalimaud Foundation, Av. Brasilia, Lisbon 1400-038, Portugal; ^3^ Max Planck Institute for Infection Biology, Berlin 10117, Germany; ^4^ Max Planck Institute for Multidisciplinary Sciences, Göttingen 37077, Germany; ^5^ Hagler Institute for Advanced Study, Texas A&M University, College Station, TX 77843, USA

**Keywords:** BCG immunotherapy, Bladder cancer, Zebrafish xenografts, Macrophages, Innate immunity, TNF signaling

## Abstract

The Bacillus Calmette–Guérin (BCG) vaccine is the oldest cancer immunotherapeutic agent in use. Despite its effectiveness, its initial mechanisms of action remain largely unknown. Here, we elucidate the earliest cellular mechanisms involved in BCG-induced tumor clearance. We developed a fast preclinical *in vivo* assay to visualize in real time and at single-cell resolution the initial interactions among bladder cancer cells, BCG and innate immunity using the zebrafish xenograft model. We show that BCG induced the recruitment and polarization of macrophages towards a pro-inflammatory phenotype, accompanied by induction of the inflammatory cytokines *tnfa*, *il1b* and *il6* in the tumor microenvironment. Macrophages directly induced apoptosis of human cancer cells through zebrafish TNF signaling. Macrophages were crucial for this response as their depletion completely abrogated the BCG-induced phenotype. Contrary to the general concept that macrophage anti-tumoral activities mostly rely on stimulating an effective adaptive response, we demonstrate that macrophages alone can induce tumor apoptosis and clearance. Thus, our results revealed an additional step to the BCG-induced tumor immunity model, while providing proof-of-concept experiments demonstrating the potential of this unique model to test innate immunomodulators.

## Teaser

**Summary:** The BCG vaccine induces bladder cancer clearance by recruiting and polarizing macrophages towards a pro-inflammatory state, directly inducing apoptosis through TNF signaling, adding an extra step to the BCG-induced tumor immunity model.

## INTRODUCTION

The Bacille Calmette–Guérin (BCG) vaccine, based on the ‘Coley's toxins’ principle, is the cancer immunotherapeutic agent that has been the longest in use (Lobo et al., 2021; Pettenati and Ingersoll, 2018; Dobosz and Dzieciątkowski, 2019). In bladder cancer, BCG is the most effective treatment to avoid disease relapse. Tumors staged as intermediate or high-risk non-muscle-invasive bladder cancer (NMIBC) are treated with intravesical BCG immunotherapy approximately 2 weeks after transurethral resection of bladder tumor. BCG induction therapy consists of six weekly instillations, after which maintenance therapy of 1 to 3 years is highly recommended to prevent progression and recurrence (Jordan and Meeks, 2019; https://uroweb.org/guidelines/non-muscle-invasive-bladder-cancer/chapter/disease-management). Despite being the gold standard of treatment of NMIBC for 40 years, BCG intravesical immunotherapy has a high rate of adverse effects, there are worldwide shortages in its supply chain, and some patients are resistant to treatment (Pettenati and Ingersoll, 2018; Dockrell and Smith, 2017; Redelman-Sidi et al., 2014). Additionally, the mechanisms through which BCG induces anti-tumor activity are not fully understood and BCG therapy has remained mostly unchanged (Lobo et al., 2021; Redelman-Sidi et al., 2014; Morales et al., 1976). Several studies have underscored the importance of a local inflammatory reaction in the bladder and a strong activation of the innate and adaptive immune systems upon BCG instillation (Lobo et al., 2021; Pettenati and Ingersoll, 2018; Redelman-Sidi et al., 2014; Prescott et al., 2000). The initial steps following instillation (∼120 min into the beginning of the treatment) have been elucidated through *in vitro* and murine studies, and not all data have been supported by human studies.

A multi-step model of BCG-induced tumor immunity has been proposed (Pettenati and Ingersoll, 2018). In steps 1 and 2, upon treatment, BCG binds to and invades the bladder lumen, interacting with the urothelium and tissue-resident macrophages. In step 3, BCG is then internalized by immune cells, notably phagocytes (Pettenati and Ingersoll, 2018; Redelman-Sidi et al., 2014; Prescott et al., 2000), and induces an innate immune response that triggers a strong local induction of pro-inflammatory cytokines and chemokines. This stimulates the recruitment of immune cells including neutrophils, monocytes, macrophages, T lymphocytes, B lymphocytes and natural killer cells. Macrophages and other antigen-presenting cells present BCG antigens to T lymphocytes through the major histocompatibility complex class II and trigger an adaptive immune response. In step 4, therapy is thought to be successful if the induction of the adaptive immune response is biased towards Th1 cells (Pettenati and Ingersoll, 2018; Redelman-Sidi et al., 2014). The recruitment of all these immune cells leads to the development of granulomatous lesions in the bladder wall (Redelman-Sidi et al., 2014; Prescott et al., 2000; van Puffelen et al., 2020).

Due to difficulties in assessing treatment response in patients, animal models of bladder cancer have been used to understand the mechanisms of BCG immunotherapy (John and Said, 2017). Historically, mice have been considered the gold-standard xenograft model owing to their highly conserved genetic likeness with humans (Ito et al., 2002; Kobayashi et al., 2015). Nevertheless, the mouse xenograft model carries some disadvantages, such as: the need for immunosuppressed or humanized animals and a large abundance of donor tumor cells (not compatible with biopsies or limited numbers of samples); long waiting times; high husbandry costs; and a moderate to low percentage of success in clinical trials. Additionally, single-cell live imaging is difficult due to their anatomical characteristics, namely, skin and fur (Kobayashi et al., 2015; Xiao et al., 2020; Wertman et al., 2016; Ellenbroek and van Rheenen, 2014).

The similarities in molecular pathways and drug responses between zebrafish and humans, and the ease in genetic manipulation have allowed for the development of robust cancer models. In zebrafish cancer xenografts, where human tumor cells are injected into zebrafish embryos or adults, cancer features such as proliferation, angiogenesis, metastasis and interactions in the tumor microenvironment (TME) can be rapidly visualized in real time and at the single-cell level due to the optical transparency of the model (Xiao et al., 2020; Wertman et al., 2016; Santoriello and Zon, 2012; Cagan et al., 2019; Stoletov and Klemke, 2008; Tulotta et al., 2016; Chapman et al., 2014; Fior et al., 2017; Poudel et al., 2018; Xue and Roh-Johnson, 2019; Yan et al., 2019). Zebrafish xenografts have helped elucidate the different chemosensitivity and radiosensitivity profiles of several cancer cell types, highlighting their importance in future personalized medicine (Fior et al., 2017; Costa et al., 2020; Tavares Barroso et al., 2021; Rebelo de Almeida et al., 2020; Varanda et al., 2020; Kowald et al., 2023). The role of the innate immune system in colorectal cancer progression and response to therapy has also been shown in this model (Poudel et al., 2018; Póvoa et al., 2021), and live imaging has allowed dissection of the earliest stages of cancer development and metastatic spread (Chapman et al., 2014; Zhao et al., 2011; Welker et al., 2017; Osmani and Goetz, 2019; Hyenne et al., 2019). Altogether, research in zebrafish cancer xenografts facilitates the rapid identification of novel cancer mechanisms that can be targeted by specific therapeutic approaches. In parallel, the zebrafish model has proven to be a powerful tool to study human tuberculosis (TB) (Davis and Ramakrishnan, 2009; Roca et al., 2019; Matty et al., 2019; Pagán and Ramakrishnan, 2018; Conrad et al., 2017; Cambier et al., 2014; Cronan et al., 2018, 2021; Varela and Meijer, 2022), in particular, for the initial mechanisms involved in the pathophysiology of TB and granuloma development (Davis and Ramakrishnan, 2009; Roca et al., 2019; Matty et al., 2019; Conrad et al., 2017; Cambier et al., 2014; Cronan et al., 2018, 2021; Behr et al., 2019). This highlights the importance of the zebrafish model in the study of the role of the innate immune system in the development of complex pathologies.

Here, we characterized part of the initial innate immune response mechanisms that occur within the TME upon BCG treatment. Using real-time single-cell-resolution microscopy, we demonstrate *in vivo* in a bladder cancer zebrafish xenograft that BCG immunotherapy induced cancer cell apoptosis and clearance of tumors through macrophages and TNF signaling. BCG stimulated a massive recruitment of macrophages that were polarized towards a Tnfa-positive pro-inflammatory phenotype. Using high-resolution live microscopy, we revealed that the presence of BCG in the TME induced profound changes in macrophage morphology and in cell–cell interactions. Innate immune cells were crucial for the anti-tumor effects of BCG, as, in their absence, tumor clearance was halted. Importantly, we demonstrate the utility of our xenografts in a preclinical setting, testing the efficacy of a newly genetically modified BCG vaccine (VPM1002 – *Mycobacterium bovis* BCGΔ*ureC*::*hly*) (Nieuwenhuizen et al., 2017; Grode, 2005) versus the conventional BCG vaccine. This next-generation BCG-based vaccine is currently undergoing three phase III efficacy trials against TB and has already shown promising effects against bladder cancer (Rentsch et al., 2022).

In summary, we dissected the earliest mechanisms of BCG immunotherapy and unveiled an additional step to the BCG-induced tumor immunity model – an active role of macrophages in the induction of tumor clearance, which had not been previously considered. Additionally, we provide proof-of-concept experiments for the use of zebrafish embryo xenografts in the preclinical setting to test new medicines aimed at boosting the innate immune response of the host, highlighting the potential of this model to become an integral part of future immunotherapy research.

## RESULTS

### The BCG vaccine induces bladder cancer clearance and apoptosis

We started by developing a xenograft bladder cancer model for BCG immunotherapy in zebrafish embryos. For this purpose, we chose two bladder cancer cell lines, one isolated from a primary tumor staged as high-risk NMIBC (NMIBC-RT112) (Rigby and Franks, 1970) and another isolated from a tumor staged as muscle-invasive bladder cancer (MIBC-J82) (O'Toole et al., 1978).

**Fig. 1.**
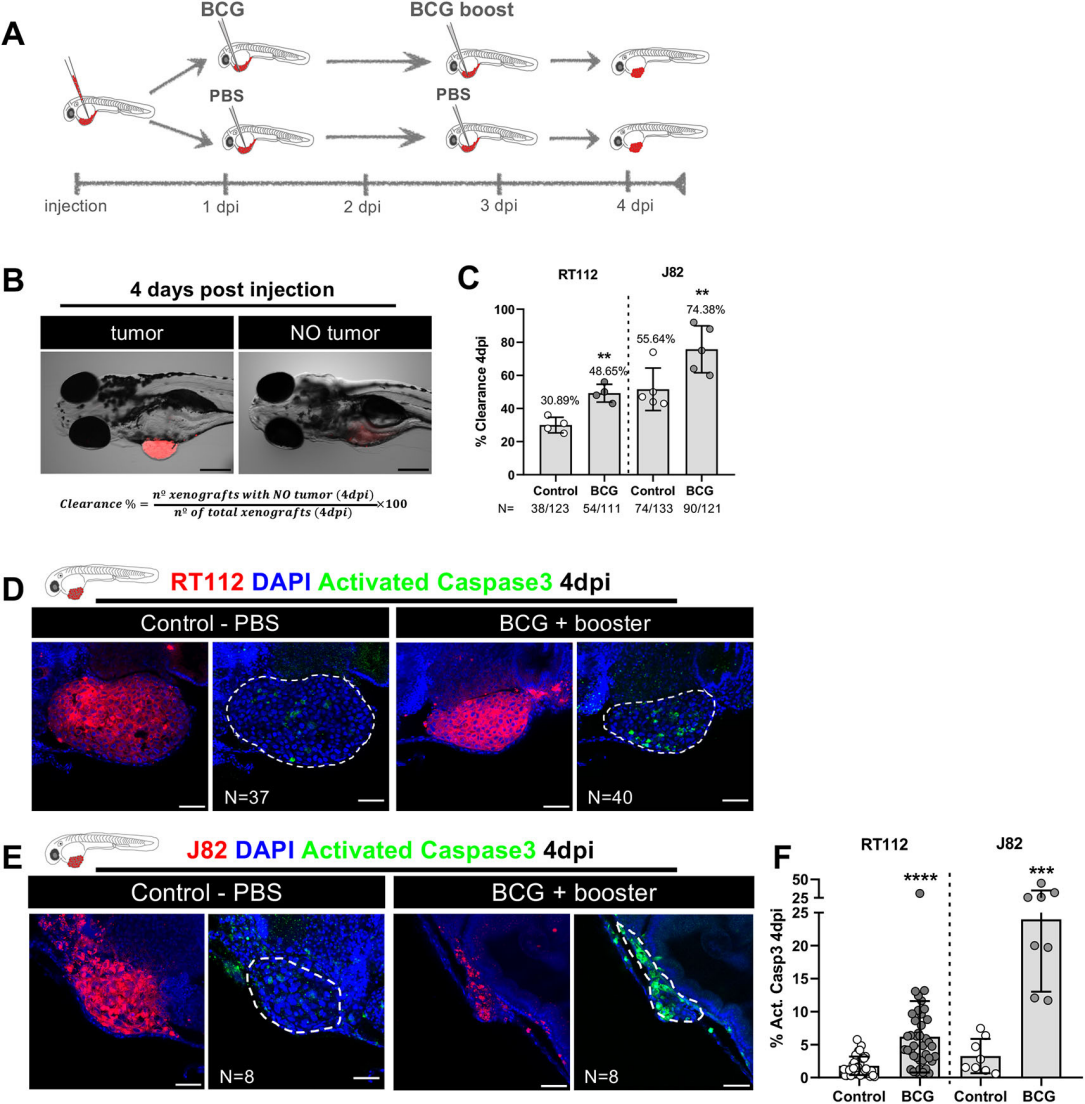
**Zebrafish bladder cancer xenografts are susceptible to BCG immunotherapy.** (A) Schematic representation of the BCG treatment protocol. (B) Representative brightfield images of xenografts with and without tumors at 4 days post injection (dpi). Human cancer cells were labelled with the Vybrant CM-DiI lipophilic stain (red) and the equation used for the calculation of clearance rate is shown. Scale bar: 250 µm. (C) Quantification of the percentage of clearance in NMIBC-RT112 and MIBC-J82 xenografts at 4 dpi. Bars indicate the results as mean±s.d. and each dot represents a full round of injections. *N* represents the number of xenografts without tumors at 4 dpi relative to the total number of xenografts at 4 dpi (**P<0.01; Fisher's exact test). (D,E) Representative confocal images of NMIBC-RT112 (D) and MIBC-J82 (E) control and BCG+booster-treated xenografts at 4 dpi. Human cancer cells were labelled with the Vybrant CM-DiI lipophilic stain (red), the apoptosis marker activated caspase-3 is in green and nuclei (DAPI counterstaining) in blue. White dashed regions outline the tumor. BCG were labelled with either the Deep Red Cell Tracker or the Vybrant CM-DiI lipophilic stains (not shown). In all images, anterior is to the left, posterior to the right, dorsal up and ventral down. Scale bars: 50 µm. (F) Quantification of the percentage of activated caspase-3-positive (apoptotic) cells to the total number of cells at 4 dpi. Bars indicate the results as mean±s.d. and each dot represents one xenograft pooled from two independent experiments. The numbers of analyzed xenografts are indicated in D,E. ***P<0.001; ****P<0.0001 (parametric unpaired two-tailed *t*-test). Note that the experiments presented in this figure and in Fig. S4 were performed in parallel; thus, they share the same set of controls and BCG+booster samples, and several transgenic backgrounds were used (see Table S1).

To optimize the BCG immunotherapy protocol and aware of the limited worldwide supply of intravesical BCG, we made use of the lyophilized BCG (OncoTICE^®^) used for patients with bladder cancer at the Day Hospital of the Champalimaud Foundation's Clinical Centre. We labeled the bacteria with a lipophilic dye to allow for their identification and prepared them for injection. To generate the bladder cancer zebrafish embryo xenografts, bladder cancer cells were fluorescently labeled with a lipophilic dye and injected into the perivitelline space (PVS) of 2 day-post-fertilization (dpf) zebrafish embryos as previously described (Fior et al., 2017; Martinez-Lopez et al., 2021). At 1 day post injection (dpi), bladder cancer xenografts were treated with one dose of intratumoral BCG, followed by a booster injection at 3 dpi and analysis on the following day (Fig. 1A). Control xenografts followed the same treatment protocol but received PBS injections instead of BCG (Fig. 1A; Fig. S1). During the first week of zebrafish development, only innate immunity is active (adaptive immunity is only mature at 2-3 weeks) (Gut et al., 2017; Soza-Ried et al., 2010; Jin et al., 2009). As our xenograft assay was performed during this first week of development, it provides an ideal temporal separation to specifically analyze the immediate effects mediated by innate immunity in the presence of cancer cells as a response to BCG treatment.

We assessed the impact of BCG treatment by evaluating *in vivo* tumor cell clearance, which was defined as the frequency of treated xenografts that lost the tumor mass at 4 dpi (Fig. 1B). Although both cell lines showed a baseline spontaneous tumor clearance – ∼30% in NMIBC-RT112 and ∼56% in MIBC-J82, BCG treatment increased the clearance efficiency in NMIBC-RT112 xenografts (1.6-fold increase, **P=0.0072). In MIBC-J82 xenografts, BCG also increased the efficiency of tumor clearance, but in a less pronounced manner (1.3-fold increase, **P=0.0076) (Fig. 1B,C). In conclusion, BCG efficiently induces bladder cancer cell clearance in the zebrafish embryo xenograft model.

The fact that BCG increased tumor clearance in the zebrafish xenografts raised the question of how human cancer cells were being cleared. We hypothesized that BCG could induce clearance either by direct cytotoxicity leading to cell death or by the stimulation of innate immune cells. To tackle this question, we evaluated activated caspase-3, which marks cells undergoing apoptosis. We found that BCG treatment induced apoptosis of bladder cancer cells (NMIBC-RT112, ****P<0.0001; MIBC-J82, ***P=0.0002) (Fig. 1D-F), which suggested that BCG treatment was promoting active clearance of cancer cells by inducing programmed cell death. However, given that some bladder cancer cell lines are susceptible to direct toxicity induced by BCG *in vitro* (Bevers et al., 2000), the question remained whether this could be a direct consequence of BCG toxicity or an active process of cancer cell elimination mediated within the host TME. Thus, we determined whether BCG is toxic to NMIBC-RT112 and MIBC-J82 tumor cell lines *in vitro*. BCG treatment did not significantly affect the survival of cultured cancer cells, as vehicle- and BCG-treated cells showed similar average cell numbers per field and similar abundance of apoptosis (Fig. S2). Thus, BCG is not directly toxic to NMIBC-RT112 and MIBC-J82 tumor cells, suggesting that the host TME could be actively involved in tumor cell death.

### BCG induces infiltration of macrophages and polarization towards a pro-inflammatory phenotype

**Fig. 2.**
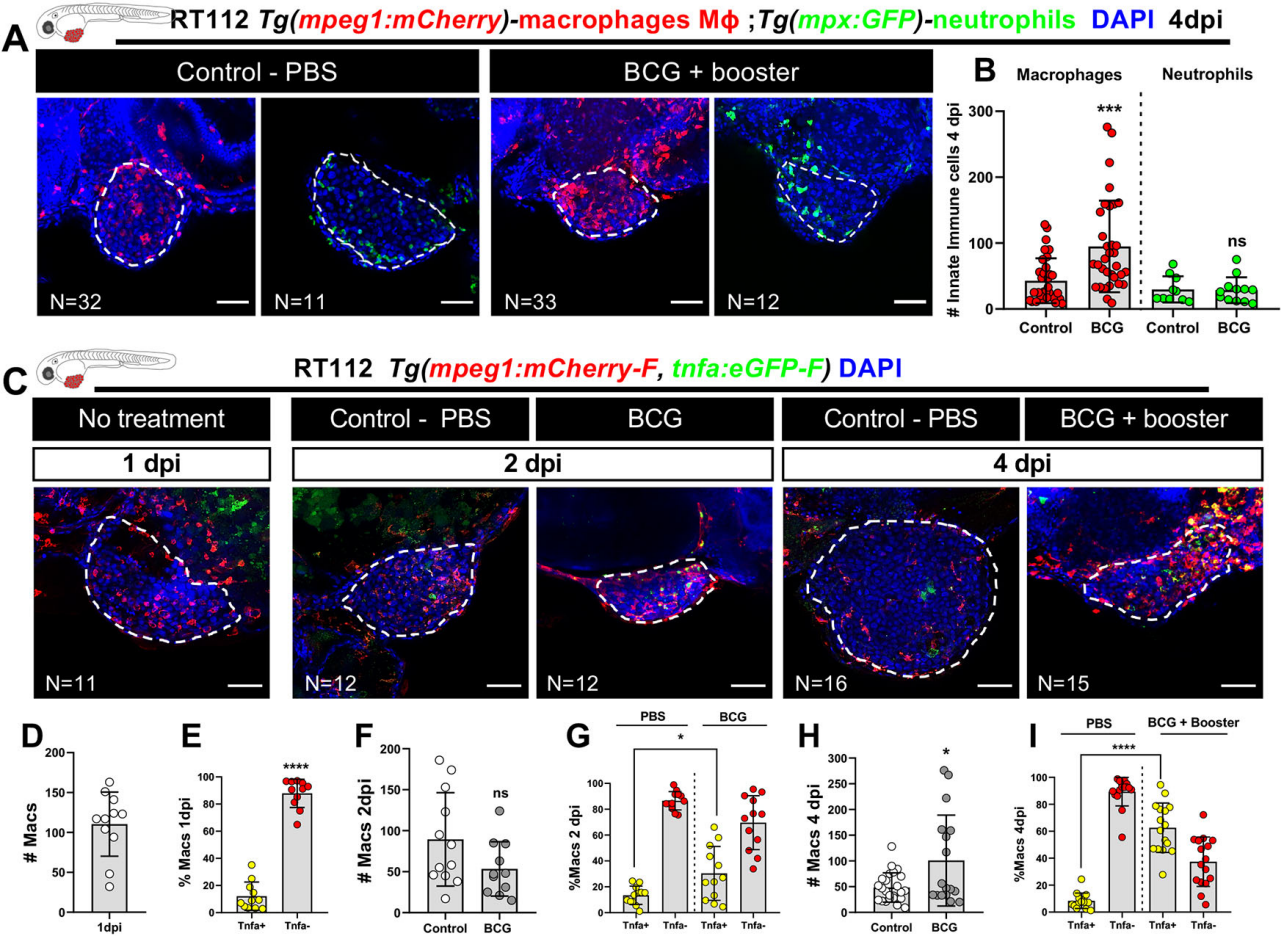
**BCG modulates recruitment and polarization of macrophages in zebrafish bladder cancer xenografts.** (A) Representative confocal images of macrophages (red) and neutrophils (green) in NMIBC-RT112 control and BCG+booster-treated xenografts, in which human cancer cells were labelled with the Deep Red Cell Tracker lipophilic stain (not shown). (B) Quantification of the absolute numbers of infiltrating macrophages and neutrophils at 4 dpi (***P=0.0003). (C) Representative confocal images of Tnfa expression (green) and macrophages (red) in NMIBC-RT112 control and BCG+booster-treated xenografts, in which human cancer cells were labelled with the Deep Red Cell Tracker lipophilic stain (not shown). In A,C, white dashed regions outline the tumor. BCG were labelled with either the Deep Red Cell Tracker or the Vybrant CM-DiI lipophilic stain (not shown). In all images, anterior is to the left, posterior to the right, dorsal up and ventral down. Scale bars: 50 µm. (D-I) Quantification of the absolute numbers of macrophages and the percentage of Tnfa-positive and Tnfa-negative macrophages in the tumor microenvironment at 1 dpi before treatment (****P<0.0001) (D,E), in control and BCG-treated xenografts at 2 dpi (ns, not significant, *P*≥0.05; *P=0.0190) (F,G), and in control and BCG+booster-treated xenografts at 4 dpi (*P<0.05; ****P<0.0001) (H,I). Bars in B,D-I indicate the results as mean±s.d. and each dot represents one xenograft pooled from two independent experiments. The numbers of analyzed xenografts are indicated in A,C. Data sets with a Gaussian distribution (B,D-I) were analyzed by parametric unpaired two-tailed *t*-test. Note that the quantitative data presented in B are also shown in Fig. 3C as these data concern the same sets of experiments and xenografts. These experiments were performed in parallel with those in Fig. 3; thus, they share the same controls, and several transgenic backgrounds were used (see Table S1).

As BCG treatment induced the elimination of human cancer cells in the zebrafish xenografts, we assumed that BCG modulates the innate response of the host embryo. Thus, to investigate this, we quantified the presence of infiltrating neutrophils and macrophages, which are the main innate immune cells at this stage of zebrafish development (Stoletov and Klemke, 2008), in bladder cancer xenografts. To this end, we injected NMIBC-RT112 bladder cancer cells into *Tg(mpx:GFP)* (Renshaw et al., 2006) and *Tg(mpeg1:mCherry)* (Renshaw et al., 2006; Ellett et al., 2011) zebrafish hosts, in which neutrophils and macrophages are fluorescently labeled, respectively (Fig. 2A). We did not detect significant differences in the absolute numbers of infiltrating neutrophils between the control and BCG-treated xenografts (Fig. 2B). In contrast, we observed a significant increase in the absolute numbers of infiltrating macrophages upon BCG treatment (numeric doubling from a mean of 47 to a mean of 97, ***P=0.0003) (Fig. 2B). Thus, these results indicate that BCG treatment induces macrophage recruitment into the TME.

**Fig. 3.**
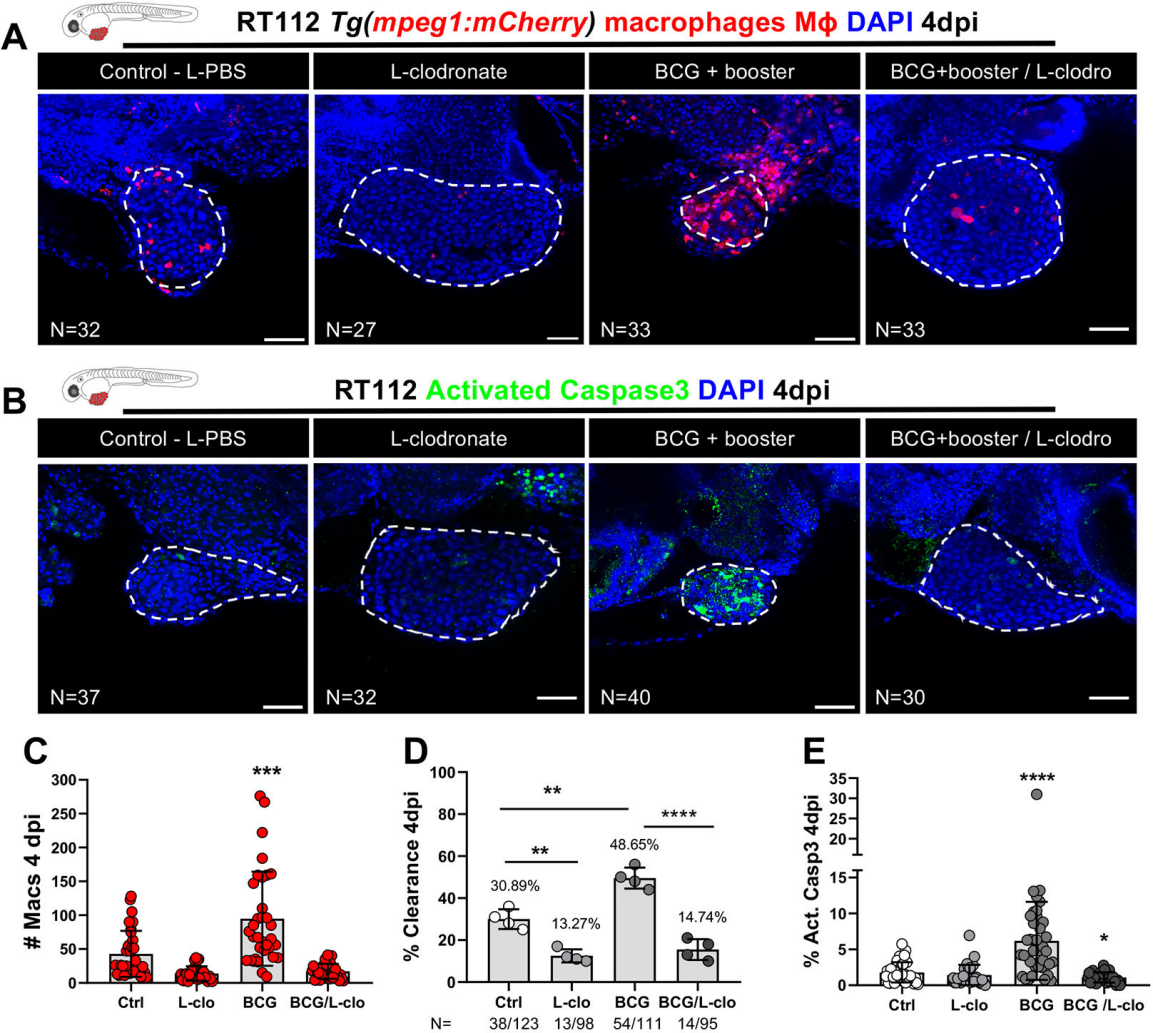
**Macrophages are essential for susceptibility of zebrafish bladder cancer xenografts to BCG immunotherapy.** (A) Representative confocal images of infiltrating macrophages (red) in BCG/L-clodronate experiments. (B) Representative confocal images of NMIBC-RT112 xenografts stained for the apoptosis marker activated caspase-3 (green) in BCG/L-clodronate experiments. In A,B, human cancer cells were labelled with the Deep Red Cell Tracker lipophilic stain (not shown), and BCG were labelled with either the Deep Red Cell Tracker or the Vybrant CM-DiI lipophilic stain (not shown). White dashed regions outline the tumor. In all images, anterior is to the left, posterior to the right, dorsal up and ventral down. Scale bars: 50 µm. (C) Quantification of the absolute numbers of infiltrating macrophages in BCG/L-clodronate experiments (***P=0.0001). Each dot represents one xenograft pooled from two independent experiments. (D) Quantification of the percentage of clearance in BCG/L-clodronate experiments at 4 dpi (**P<0.01; ****P<0.0001; Fisher's exact test). Each dot represents a full round of injections and *N* represents the number of xenografts without tumors at 4 dpi relative to the total number of xenografts at 4 dpi. (E) Quantification of the percentage of activated caspase-3-positive (apoptotic) cells in BCG/L-clodronate experiments at 4 dpi (*P=0.0102; ****P<0.0001). Each dot represents one xenograft pooled from three independent experiments*.* Bars indicate the results as mean±s.d. and the numbers of analyzed xenografts are indicated in A,B. Data sets that did not pass the D'Agostino–Pearson omnibus and Shapiro–Wilk normality tests were analyzed by nonparametric unpaired Mann–Whitney test (C,E). Unless stated otherwise, each experimental data set was challenged to the respective control. Additionally, the data sets in C,E were analyzed with Welch's one-way ANOVA with Games–Howell post hoc test in which *P*<0.0001. Xenografts represented in A,B correspond to the same sets of experiments and genetic background in which transgenic larvae were also labeled for activated caspase-3. Quantitative data shown in C are also shown in Fig. 2B as these data concern the same sets of experiments and xenografts. The experiments in this figure were performed in parallel with those in Fig. S5; thus, they share the same controls. Note that several transgenic backgrounds were used (see Table S1).

**Fig. 4.**
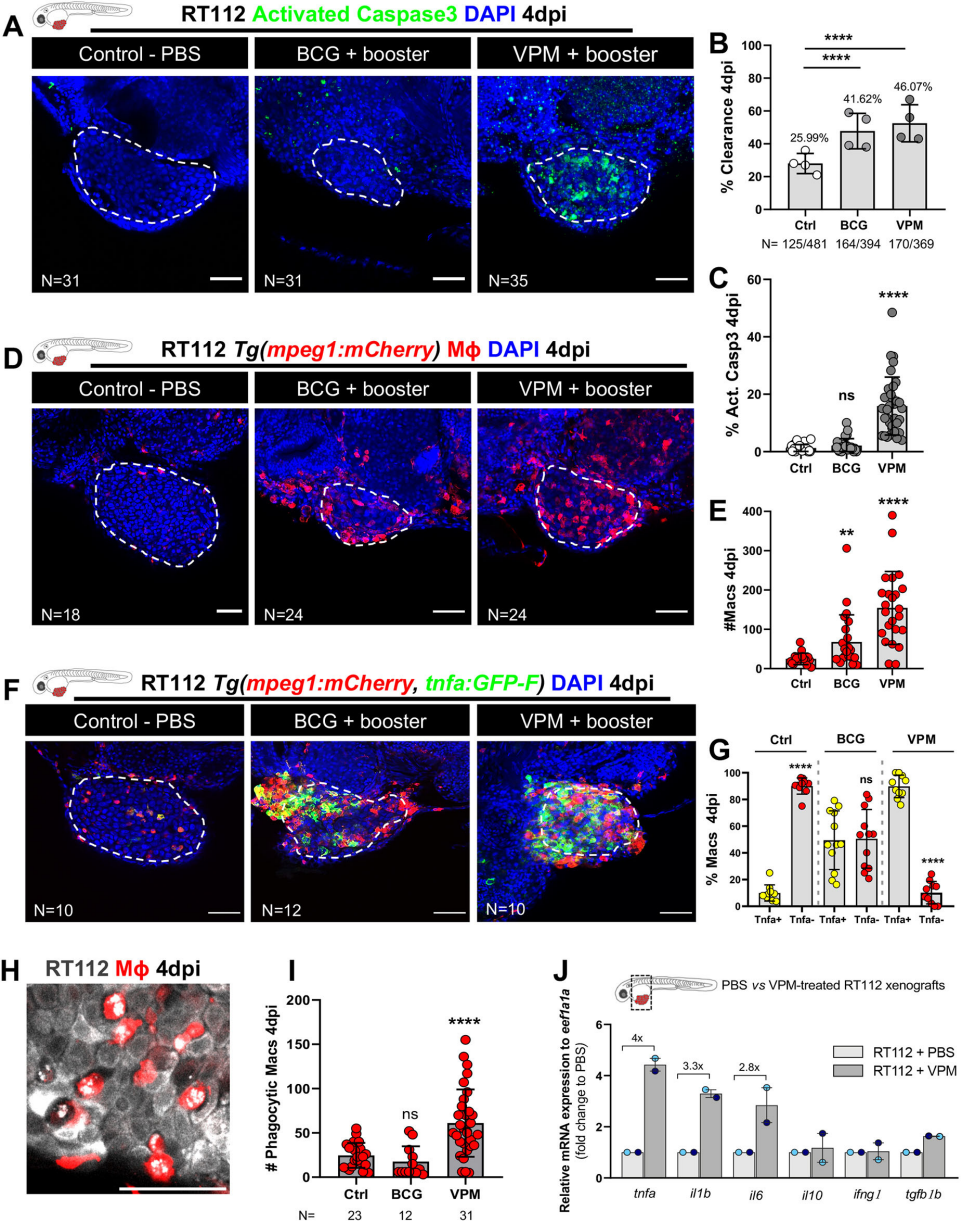
**Zebrafish bladder cancer xenografts are susceptible to immunotherapy with the conventional and genetically modified BCG strains.** (A) Representative confocal images of NMIBC-RT112 control and BCG+booster-treated or VPM1002+booster-treated xenografts. Human cancer cells were labelled with the Deep Red Cell Tracker lipophilic stain (not shown) and were stained for the apoptosis marker activated caspase-3 (green) at 4 dpi. (B) Quantification of the percentage of clearance in NMIBC-RT112 control and treated xenografts at 4 dpi (****P<0.0001; Fisher's exact test). Each dot represents a full round of injections in which *N* represents the number of xenografts without tumors at 4 dpi relative to the total number of xenografts at 4 dpi. (C) Quantification of the percentage of activated caspase-3-positive (apoptotic) cells in NMIBC-RT112 control and treated xenografts at 4 dpi (****P<0.0001). (D) Representative confocal images of infiltrating macrophages (red) in NMIBC-RT112 control and treated xenografts at 4 dpi. (E) Quantification of absolute numbers of infiltrating macrophages in NMIBC-RT112 control and treated xenografts at 4 dpi (**P=0.0032; ****P<0.0001). (F) Representative confocal images of Tnfa expression (green) and macrophages (red) in NMIBC-RT112 control and treated xenografts at 4 dpi. Human cancer cells were labelled with the Deep Red Cell Tracker lipophilic stain (not shown). In A,D,F, white dashed regions outline the tumor. BCG+booster-treated and VPM1002+booster-treated xenografts were labelled with either the Deep Red Cell Tracker or the Vybrant CM-DiI lipophilic stain (not shown). In all images, anterior is to the left, posterior to the right, dorsal up and ventral down. Scale bars: 50 µm. (G) Quantification of the percentage of Tnfa-positive and Tnfa-negative macrophages in the tumor microenvironment (TME) of NMIBC-RT112 control and BCG+booster-treated or VPM1002+booster-treated xenografts at 4 dpi (****P<0.0001). Each dot represents one xenograft pooled from two independent experiments. (H) Representative confocal image of macrophages (red) and NMIBC-RT112 cells labelled with the Deep Red Cell Tracker lipophilic stain. (I) Quantification of the number of phagocytic macrophages in NMIBC-RT112 control and treated xenografts at 4 dpi (****P<0.0001). Each dot represents one xenograft pooled from two independent experiments. (J) Relative gene expression levels of zebrafish *tnfa*, *il1b*, *il6*, *il10*, *ifng1* and *tgfb1b* at 4 dpi in the TME of NMIBC-RT112 control and VPM1002+booster-treated xenografts. Bars indicate the fold change of expression to that in the control relative to expression of the housekeeping gene. Each dot represents the average of two or three technical replicates of one independent experiment. In B,C,E,G,I,J, bars indicate the results as mean±s.d. For C,E,G, the numbers of analyzed xenografts are indicated in A,D,F. Data sets with a Gaussian distribution (G) were analyzed by parametric unpaired two-tailed *t*-test, and data sets that did not pass the D'Agostino–Pearson omnibus and Shapiro–Wilk normality tests were analyzed by nonparametric unpaired Mann–Whitney test (C,E). Unless stated otherwise, each experimental data set was challenged to the respective control. Additionally, the data sets in C,E,I were analyzed with Welch's one-way ANOVA with Games–Howell post hoc test in which *P*<0.0001, *P=*0.0005 and *P*<0.0001, respectively. ns, not significant, *P*≥0.05. Note that the quantification presented in E is also shown in Fig. 7F, as these data concern the same sets of experiments and xenografts. Data from J are used for the mRNA expression comparison shown in Fig. 7H. Note that several transgenic backgrounds were used (see Table S1).

Notably, although macrophage recruitment to the TME shows activation of the immune system by BCG, macrophage recruitment does not inform whether the macrophages contribute to the elimination of human cancer cells. This is because macrophages can adopt either a pro-inflammatory (M1-like) or anti-inflammatory (M2-like) phenotype with tumor-suppressing or tumor-promoting functions, respectively (Pittet et al., 2022; Mantovani, 2009; Galdiero et al., 2013; Keeley et al., 2019). Thus, to investigate whether BCG modulates macrophage polarization towards a pro-inflammatory M1-like state, we analyzed the presence of Tnfa-producing macrophages, which are considered M1-like with tumor-suppressing functions. For this, we generated bladder cancer xenografts in double-transgenic zebrafish carrying a general macrophage mCherry reporter driven by the *mpeg1* promoter and a GFP reporter driven by the *tnfa* promoter [*Tg*(*mpeg1:mCherry-F; tnfa:eGFP-F*)] (Nguyen-Chi et al., 2017)*.* Infiltrating macrophages were analyzed at 1 dpi (Fig. 2C-E), 2 dpi (Fig. 2C,F,G) and 4 dpi (Fig. 2C,H,I). Quantification of the immune cell populations revealed that at 1 dpi, prior to treatment, macrophages were mostly Tnfa negative. However, upon BCG treatment, macrophages gradually polarized towards a Tnfa-positive pro-inflammatory phenotype and, at 4 dpi, Tnfa-positive macrophages represented ∼62% of the total macrophage population in the tumors of BCG-treated xenografts, whereas in the controls, they represented only ∼8% (****P<0.0001) (Fig. 2C,H,I). In addition, BCG treatment also induced a change in macrophage morphology from a mesenchymal or dendritic-like morphology to an ameboid and vacuole-rich morphology (Fig. S3A-D). These results suggest that tumor elimination driven by BCG treatment is mediated by pro-inflammatory macrophages with tumor-suppressing activity.

### Macrophages mediate BCG-induced tumor clearance

BCG treatment activated an anti-tumor response by inducing clearance and apoptosis with a strong recruitment of macrophages and their polarization towards a Tnfa-expressing M1-like phenotype in zebrafish xenografts, which suggested that macrophages play a critical role in this response. To test this, we pharmacologically depleted macrophages by using liposomes containing clodronate (referred to as L-clodronate or L-clodro), which are selectively phagocytosed by macrophages. For this, we injected 0.07 µg of L-clodronate intratumorally at the same timepoints described in Fig. 1A. Quantification of macrophages confirmed that L-clodronate efficiently reduced the number of macrophages in the TME and almost completely abrogated their local presence (Fig. 3A,C). Remarkably, the anti-tumor effects of BCG, namely, induction of tumor clearance and apoptosis, were fully abolished upon macrophage depletion (Fig. 3B,D,E). The same phenotype was observed in MIBC-J82 xenografts (Fig. S4). Interestingly, when comparing the liposome-encapsulated PBS (L-PBS) controls to L-clodronate-treated xenografts that did not receive BCG treatment, we observed that the depletion of macrophages resulted in reduction of spontaneous clearance (Fig. 3D). We conclude that bladder cancer tumor cells are spontaneously eliminated by macrophages and that BCG treatment profoundly elevates their tumor clearance activity.

To rule out that the macrophage-dependent effect of BCG is an artifact of the zebrafish bladder cancer xenografts, we treated NMIBC-RT112 xenografts with the cytotoxic drug mitomycin C (https://uroweb.org/guidelines/non-muscle-invasive-bladder-cancer/chapter/disease-management). As expected, mitomycin C exerted its anti-tumor cytotoxic effect even in the absence of macrophages (Fig. S5). Taken together, these findings revealed that the initial tumor clearance and induction of apoptosis upon BCG immunotherapy is mediated by macrophages that are recruited to the bladder tumor. The mode of action of BCG in this model is through the innate immune system and not through direct BCG toxicity on the cancer cells.

### VPM1002 is more efficient in inducing tumor clearance and a pro-inflammatory TME than the conventional BCG vaccine

We next tested the tumor-suppressing efficiency of the standard BCG vaccine in comparison with a novel promising next-generation vaccine candidate of BCG, the VPM1002 vaccine (*M. bovis* BCGΔ*ureC*::*hly*) (Lobo et al., 2021; Pettenati and Ingersoll, 2018; Grode, 2005; Lalvani and Sridhar, 2010; Kaufmann, 2020). VPM1002 is a genetically modified BCG vaccine strain. In this strain, the urease C-encoding gene (*ureC*) was replaced by the listeriolysin O-encoding gene (*hly*). *hly* is derived from *Listeria monocytogenes*, and the main role of listeriolysin O is to disrupt the phagosomal membrane provided that the phagosomal milieu is acidic. This genetic modification confers the VPM1002 strain with higher immunogenicity by allowing mycobacterial antigens to escape to the cytosol of macrophages. Moreover, membrane perturbation allows egress of double-stranded DNA, which induces inflammasome activation, resulting in generation of IL-1β and IL-18, as well as induction of LC3-II as a marker for autophagy and xenophagy (Kaufmann, 2020). VPM1002 is currently undergoing three phase III clinical efficacy trials to assess its efficacy in TB prevention in different populations in sub-Saharan Africa and India (https://clinicaltrials.gov/study/NCT03152903; https://clinicaltrials.gov/study/NCT04351685; https://newtbvaccines.org/vaccine/immuvac/). A phase II clinical trial has also been performed to evaluate its effects in bladder cancer treatment in Switzerland and Germany (Nieuwenhuizen et al., 2017; Grode, 2005; Rentsch et al., 2022; Kaufmann, 2020; Saiga et al., 2015; Gengenbacher et al., 2016; https://clinicaltrials.gov/ct2/show/NCT04439045).

Thus, we generated working stocks from live cultures of conventional BCG and VPM1002 and injected the bladder cancer xenografts with BCG and VPM1002 (Rentsch et al., 2022) intratumorally. We followed the same treatment schedule shown in Fig. 1A. We chose BCG:SSI as the control strain due to its genetic profile, which is closer to that of VPM1002 (Bottai and Brosch, 2016). Our results show that both conventional BCG and VPM1002 strains were able to induce ∼45% of tumor clearance (Fig. 4A,B). However, and in alignment with previous studies (Nieuwenhuizen et al., 2017; Saiga et al., 2015), VPM1002 induced a significantly higher infiltration of macrophages and more pronounced tumor apoptosis in the TME than the conventional BCG vaccine (****P<0.0001) (Fig. 4A,C-E). With regards to neutrophil infiltration, we could not detect significant changes between the two vaccines (Fig. S6).

The conventional BCG vaccine polarized macrophages towards a pro-inflammatory phenotype at 4 dpi (from ∼11% of Tnfa-positive macrophages in controls to ∼50% in BCG-treated xenografts), but the VPM1002 vaccine was much more efficient in generating a highly pro-inflammatory TME with ∼90% of the macrophages being Tnfa positive (****P<0.0001) (Fig. 4F,G). In addition, VPM1002 also induced a significant increase in the number of macrophages engaged in phagocytosis (****P<0.0001) (Fig. 4H,I).

To confirm the induction of a pro-inflammatory TME by VPM1002 treatment, we measured the relative gene expression of zebrafish *tnfa*, *il1b*, *il6*, *il10*, *ifng1* and *tgfb1b* at 4 dpi in the tumors of control and VPM1002-treated NMIBC-RT112 xenografts (Fig. 4J). VPM1002 induced the expression of the pro-inflammatory cytokines *tnfa* (4-fold increase), *il1b* (3.3-fold increase) and *il6* (2.8-fold increase) relative to that in the control xenografts, further suggesting that VPM1002 treatment induces a highly inflammatory TME (Fig. 4J). These results are in agreement with inflammatory phenotypes described in macrophages *in vitro* and in mice after VPM1002 exposure (Nieuwenhuizen et al., 2017; Saiga et al., 2015).

### BCG and VPM1002 vaccines stimulate macrophage kinetics and their intercellular interactions

**Fig. 5.**
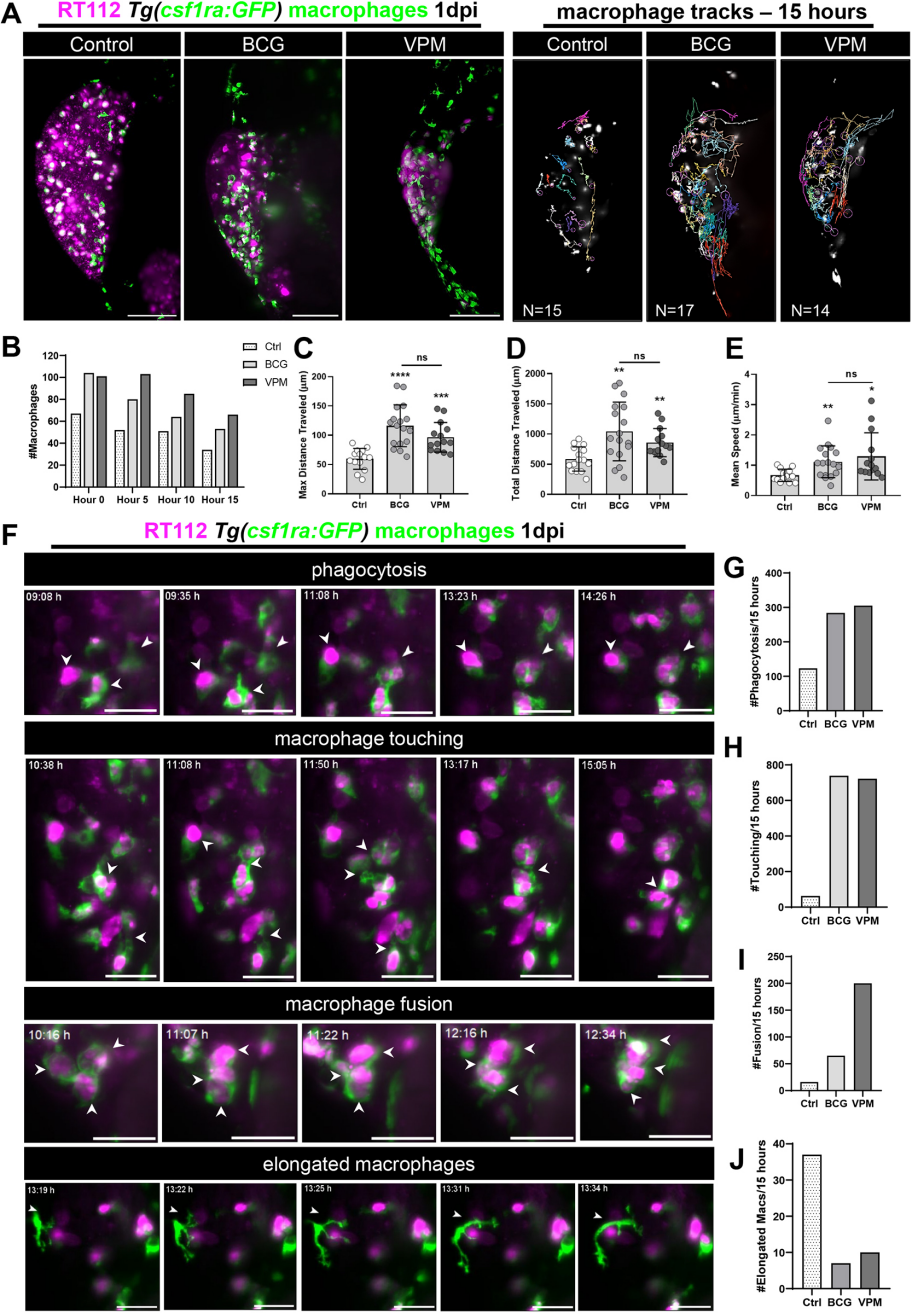
**Live imaging reveals that BCG and VPM1002 vaccines stimulate macrophage kinetics and their intercellular interactions.** (A) Left: representative maximum-intensity projections of NMIBC-RT112 cells labelled with the Deep Red Cell Tracker lipophilic stain (magenta) and for macrophages (green) at 15 h of light-sheet imaging. RT112 xenografts were imaged at 1 dpi right after BCG and VPM1002 injection. Right: representation of the macrophage tracks in which each colored line shows the path that an individual macrophage followed throughout 15 h. Scale bars: 100 µm. (B) Quantification of the absolute numbers of macrophages in NMIBC-RT112 control and BCG- or VPM1002-treated xenografts at different timepoints during imaging. (C) Quantification of the maximum distance travelled in micrometers (µm) by macrophages during 15 h after treatment in NMIBC-RT112 xenografts (***P=0.0002; ****P<0.0001). (D) Quantification of the total distance travelled in micrometers (µm) by macrophages during 15 h after treatment in NMIBC-RT112 xenografts (BCG, **P=0.0019; VPM, **P=0.0024). (E) Quantification of the mean speed in micrometers (µm) per minute travelled by macrophages during 15 h after treatment in NMIBC-RT112 xenografts (*P=0.0109; **P=0.0041). (F) Representative still images of light-sheet movies illustrating different macrophage interaction events. White arrowheads indicate each event analyzed. Scale bars: 25 µm. (G-J) Quantification of the number of phagocytic macrophages (G), the number of membrane touching events (H), the number of fusion events (I) and the number of elongated macrophages (J) observed in 15 h of imaging in NMIBC-RT112 xenografts. Bars indicate the results as mean±s.d. and each dot represents one macrophage. The numbers of analyzed xenografts are indicated in A. Data sets with a Gaussian distribution (C-E) were analyzed by parametric unpaired two-tailed *t*-test. Unless stated otherwise, each experimental data set was challenged to the respective control. Additionally, C-E were analyzed with Welch's one-way ANOVA with Games–Howell post hoc test in which *P*<0.0001, *P*=0.0008 and *P*=0.0019, respectively. ns, not significant, *P*≥0.05.

We next used light-sheet imaging to further understand how macrophages respond to the conventional BCG and VPM1002 vaccines and provide real-time visualization with single-cell resolution of these processes. At 1 dpi, immediately after treatment, control, BCG-treated and VPM1002-treated *Tg(csf1ra:GFP)* (Dee et al., 2016) xenografts, in which macrophages were fluorescently labelled in green, were imaged for 15 consecutive hours to assess the macrophage kinetics during this process (Fig. 5A; Movies 1-3). Throughout the assay, the number of macrophages in the TME of the BCG- and VPM1002-treated xenografts was higher than that in the control xenografts (Fig. 5B). Quantification of the overall movement, distance travelled and speed of macrophages revealed that these parameters were higher in both vaccine-treated conditions compared to those measured in the control (Fig. 5C-E). Additionally, BCG or VPM1002 treatment induced changes in the behavior of macrophages and their interaction with surrounding macrophages (Fig. 5F-J). BCG and VPM1002 treatment increased not only cancer cell phagocytosis (Fig. 5G), but also the frequency of macrophage membrane touching (Fig. 5H; Movie 4) and fusion events (i.e. macrophages touching membranes and fusing them) (Fig. 5I; Movie 5). Interestingly, we noticed that elongated macrophages with no phagocytic capacity (dendritic like) were more prevalent in control xenografts than in BCG- or VPM1002-treated xenografts (Fig. 5J; Movie 6). Both vaccines induced similar macrophage behaviors, with VPM1002 inducing more fusion events than the conventional BCG (Fig. 5G-J). These fusion events are reminiscent of the initiation of granuloma-like structures (Pagán and Ramakrishnan, 2018).

Overall, these results show that the presence of BCG and VPM1002 in the TME generates an instantaneous mobile response in macrophages that migrate towards tumor cells. Phagocytic macrophages constantly and closely interact with each other. This process highlights the importance of cell–cell interactions in the BCG vaccine-mediated tumor clearance.

### The BCG vaccine induces myelopoiesis

**Fig. 6.**
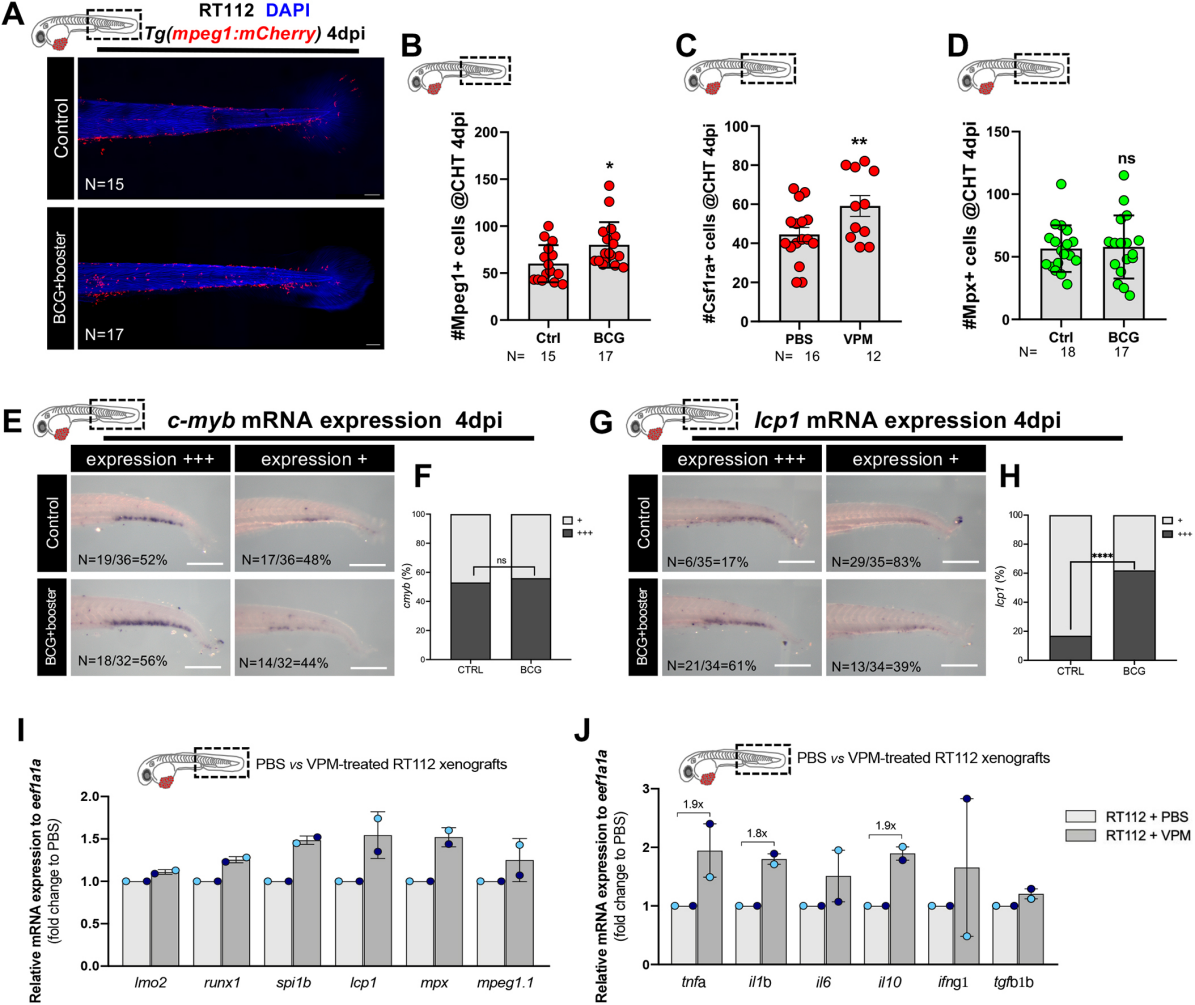
**BCG induces myelopoiesis in zebrafish bladder cancer xenografts.** (A) Representative confocal images of macrophages (red) in the caudal hematopoietic tissue (CHT) of NMIBC-RT112 control and BCG+booster-treated xenografts at 4 dpi. Scale bars: 50 µm. (B-D) Quantification of the absolute numbers of Mpeg1^+^ cells (*P=0.0155) (B), Csf1ra^+^ cells (**P=0.0064) (C) and Mpx^+^ cells (*P*=0.8611) (D) in the CHT of NMIBC-RT112 control and BCG+booster-treated xenografts at 4 dpi. Data are from one independent experiment. Bars indicate the results as mean±s.d. and each dot represents one xenograft. (E,F) mRNA expression of *c-myb* in the CHT of NMIBC-RT112 control and BCG+booster-treated xenografts at 4 dpi (E) and its corresponding quantification (F). Data were pooled from two independent experiments. (G,H) mRNA expression of *lcp1* (*l-plastin*) in the CHT of NMIBC-RT112 control and BCG+booster-treated xenografts at 4 dpi (G) and its corresponding quantification (****P<0.001; Fisher's exact test) (H). Data are from one independent experiment. For E-H, the numbers of analyzed xenografts are indicated in the images. Scale bars: 250 µm. (I,J) Relative gene expression levels of zebrafish *lmo2*, *runx1*, *spi1b*, *lcp1*, *mpx* and *mpeg1.1* (I) and *tnfa*, *il1b*, *il6*, *il10*, *ifng1* and *tgfb1b* (J) at 4 dpi in the CHT of NMIBC-RT112 control and VPM1002+booster-treated xenografts. Bars indicate the fold change of expression to that in the control relative to housekeeping gene expression (mean±s.d.). Each dot represents the average of two or three technical replicates of one independent experiment. Data sets with a Gaussian distribution (B-D) were analyzed by parametric unpaired two-tailed *t*-test. Unless stated otherwise, each experimental data set was challenged to the respective control. ns, not significant, *P*≥0.05. In all images, anterior is to the left, posterior to the right, dorsal up and ventral down. Note that several transgenic backgrounds were used (see Table S1).

It has been shown that BCG induces epigenetic changes in the hematopoietic compartment of human volunteers. These changes result in the skewing of hematopoietic stem cells towards myelopoiesis (Arts et al., 2018). Thus, we assessed whether we could also observe changes in the hematopoietic progenitors of the zebrafish xenografts upon BCG treatment. We quantified the number of macrophages and neutrophils in the caudal hematopoietic tissue (CHT) at 4 dpi (Fig. 6A-D), where hematopoiesis and myelopoiesis actively occur (Davidson and Zon, 2004). We used two transgenic reporters for macrophages, *Tg(mpeg1:mCherry)* and *Tg(csf1ra:GFP)*, and for neutrophils, we used *Tg(mpx:GFP)*. Our results show that the injection of BCG or VPM1002 led to an increase of the number of Mpeg1- and Csf1ra-positive cells in the CHT (Fig. 6A-C). However, we could not detect an increase in the numbers of Mpx-positive cells (Fig. 6D).

Next, we performed *in situ* hybridization for the early hematopoietic marker *c-myb* (also known as *myb*) (Fig. 6E,F) and the myeloid marker *lcp1* (also known as *l-plastin*) (Fig. 6G,H). Our results show that BCG specifically stimulates myelopoiesis (*lcp1*) and not general hematopoiesis (*c-myb*) (Soza-Ried et al., 2010; Davidson and Zon, 2004) (Fig. 6E-H). To further confirm these results, we dissected the tail region of control (PBS-treated) and VPM1002-treated NMIBC-RT112 xenografts and analyzed the expression of several key regulators of hematopoiesis by real-time quantitative PCR (RT-qPCR). We tested the expression of *lmo2*, *runx1*, *spi1b* (also known as *pu.1*), *lcp1*, *mpx* and *mpeg1.1*. The genes *lmo2*, *runx1* and *spi1b* are all expressed by hematopoietic stem cells, with *lmo2* and *runx1* expressed even earlier in the early hemogenic endothelium (Chen and Zon, 2009; de Jong and Zon, 2005; Farrell et al., 2018; Sur et al., 2023). *runx1* and *spi1b* later engage in a negative feedback loop that governs the equilibrium between distinct myeloid fates (Jin et al., 2012). *mpeg1.1* and *mpx* are markers of macrophages and neutrophils, respectively (Renshaw et al., 2006; Ellett et al., 2011).

Overall, our results suggest that VPM1002 does not induce general hematopoiesis at the level of the hematopoietic stem cells (no impact on *lmo2* or *c-myb* expression, Fig. 6E,F,I) but induces the expression of myeloid lineage markers. This myeloid skewing can possibly be at the level of hematopoietic myeloid stem cells (upregulation of *runx1* and *spi1b*, Fig. 6I) and/or at the level of the myeloid progenitor differentiation (upregulation of *lcp1*, *mpx* and *mpeg1.1*, Fig. 6I). To truly pinpoint which progenitor or stem cells are being induced, single-cell RNA-sequencing data would be necessary. Nevertheless, our data clearly suggest a skewing towards the myeloid compartment, in particular, towards the macrophage cell lineage (high *spi1b*). In addition, this myeloid skewing in the CHT was accompanied by a systemic induction of inflammatory cytokines (Fig. 6J) involved in myelopoiesis and macrophage differentiation (Maltby et al., 2014; Jahandideh et al., 2020).

### A robust innate immune response requires both the presence of cancer cells and the BCG vaccine

**Fig. 7.**
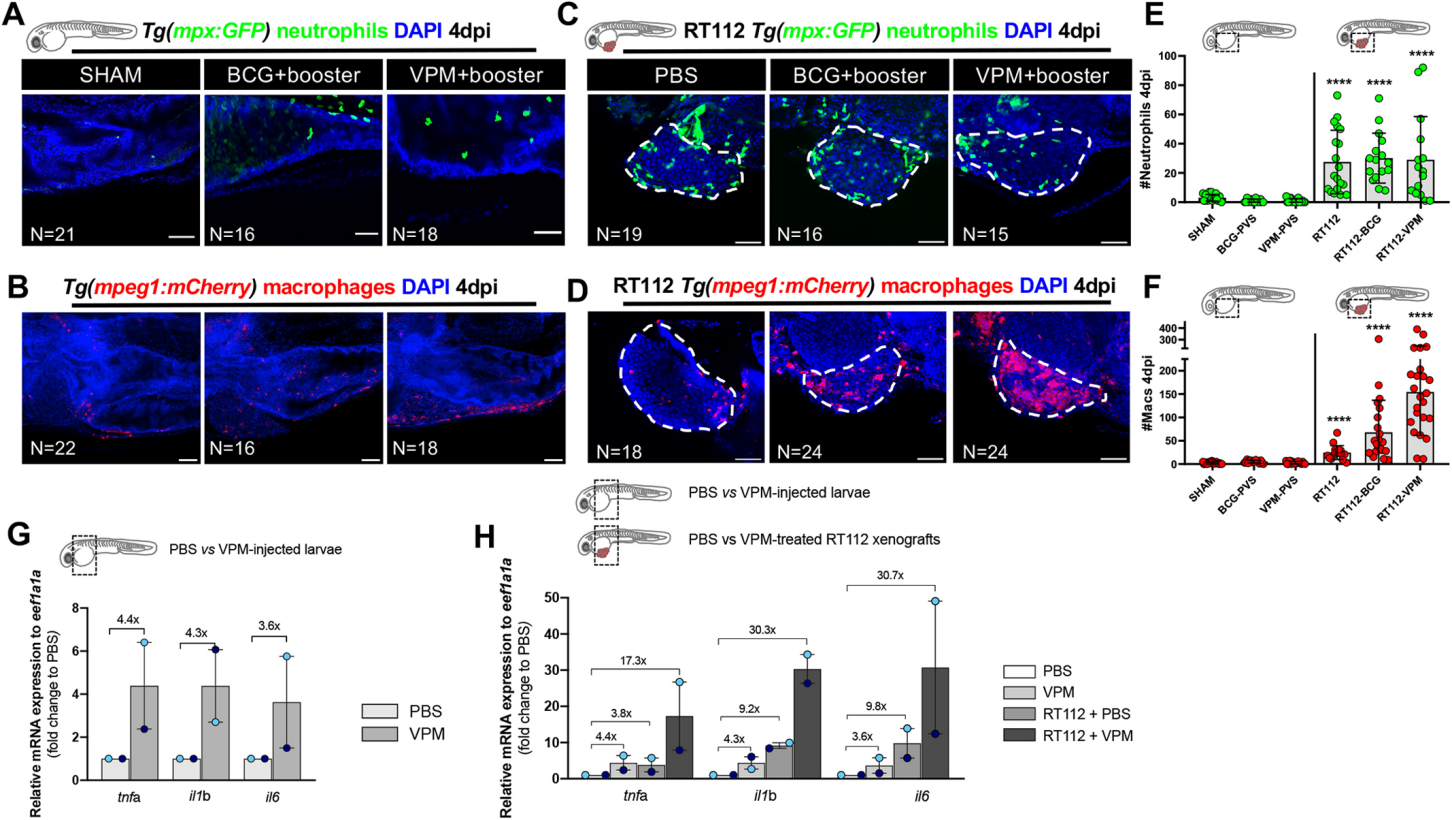
**Bladder cancer cells are required for the recruitment of neutrophils and macrophages to the PVS in response to BCG immunotherapy.** (A-D) Representative confocal images of neutrophils (green) and macrophages (red) of non-injected zebrafish larvae (A,B) and NMIBC-RT112 xenografts (C,D) at 4 dpi, in which BCG or VPM1002 were labelled with the Deep Red Cell Tracker lipophilic stain (not shown). White dashed regions outline the tumor. In all images, anterior is to the left, posterior to the right, dorsal up and ventral down. Scale bars: 50 µm. (E) Quantification of the absolute numbers of neutrophils of zebrafish larvae at 4 dpi (****P<0.0001). Note that the xenograft data shown here are also shown in Fig. S6 for analysis of the effects of BCG on neutrophil infiltration. (F) Quantification of the absolute numbers of macrophages of zebrafish larvae at 4 dpi (****P<0.0001). Neutrophil and macrophage data sets were compared against their corresponding sham control. Bars indicate the results as mean±s.d. and each dot represents one xenograft pooled from three independent experiments*.* Note that the xenograft data shown here for comparison are also shown in Fig. 4E, as these data concern the same sets of experiments/xenografts. (G) Relative gene expression levels of zebrafish *tnfa*, *il1b* and *il6* at 6 days post fertilization (dpf) in the trunk region of non-injected zebrafish larvae (control versus VPM1002+booster). (H) Relative gene expression levels of zebrafish *tnfa*, *il1b* and *il6* at 6dpf/4 dpi in the trunk region of non-injected zebrafish larvae (control versus VPM1002+booster injected) or NMIBC-RT112 xenografts (control versus VPM1002+booster), considering PBS-treated larvae as the basal control to normalize values. This graph includes data also presented in Fig. 4J. Bars in G,H indicate the fold change of expression to that in the control relative to housekeeping gene expression (mean±s.d.). Each dot represents the average of two or three technical replicates of one independent experiment. Data sets did not pass the D'Agostino–Pearson omnibus and Shapiro–Wilk normality tests were analyzed by nonparametric unpaired Mann–Whitney test (E,F). Each experimental data set was challenged to the sham control. Note that several transgenic backgrounds were used (see Table S1).

Next, we interrogated whether the strong innate immune response to BCG and VPM1002 immunotherapy was towards the bacteria alone or dependent on the presence of bladder cancer cells. Thus, we challenged embryos without cancer cells to both vaccine strains and quantified the numbers of innate immune cells (Fig. 7A-D). Surprisingly, the absolute number of immune cells (macrophages and neutrophils) in the PVS of vaccine-only-treated embryos that were not carrying bladder cancer cells was similar to that in the PBS controls, whereas when tumor cells were present, macrophages and neutrophils were recruited to the TME (Fig. 7A-D). Additionally, upon whole-body analysis, we could not see any significant differences in the numbers of macrophages and neutrophils (Fig. S7A-D). Along this line, the majority of macrophages in the vaccine-only-treated embryos displayed a similar Tnfa phenotype to that in the control embryos (Fig. S7E,F).

To further confirm these results, we dissected the PVS region of PBS- and VPM1002-injected zebrafish larvae (with no tumor cells), as well as PBS- and VPM1002-treated NMIBC-RT112 xenografts, and analyzed the expression of several key cytokines by RT-qPCR. Although there was no difference in the number of recruited innate immune cells when larvae were exposed to BCG and VPM1002 (Fig. 7E,F), we could detect an induction of gene expression of the inflammatory cytokines *tnfa* (4.4-fold increase), *il1b* (4.3-fold increase) and *il6* (3.6-fold increase) with the sole administration of VPM1002 (Fig. 7G). However, this induction was clearly enhanced by the presence of tumor cells (*tnfa*, 17.3-fold increase; *il1b*, 30.3-fold increase; *il6*, 30.7-fold increase) (Fig. 7H).

Overall, these results indicate that the sole administration of BCG or VPM1002 triggers a mild inflammatory response in the zebrafish larvae. In contrast, although injection of cancer cells alone already induced a mild recruitment of neutrophils and macrophages to the PVS (Fig. 7A-F), treatment with the conventional BCG (****P≤0.0001) and VPM1002 (****P<0.0001) vaccines induced a more profound recruitment of macrophages into the PVS region and a massive induction of inflammatory cytokines (Fig. 7H), generating an inflammatory TME. Altogether, these experiments suggest that a robust innate immune response requires both the presence of cancer cells and vaccine treatment to boost the infiltration and polarization towards a pro-inflammatory profile of macrophages in the TME, which then leads to tumor clearance.

### Tnfa signaling is essential for macrophage-mediated anti-tumor activity

Our L-clodronate experiments showed that the BCG anti-tumoral effect, clearance and apoptosis induction is macrophage dependent and that macrophages polarize towards a Tnfa-positive phenotype. This led us to hypothesize that macrophages could induce cancer cell apoptosis through TNF signaling, given the induction of Tnfa expression in macrophages upon BCG treatment (Fig. 4F,G).

**Fig. 8.**
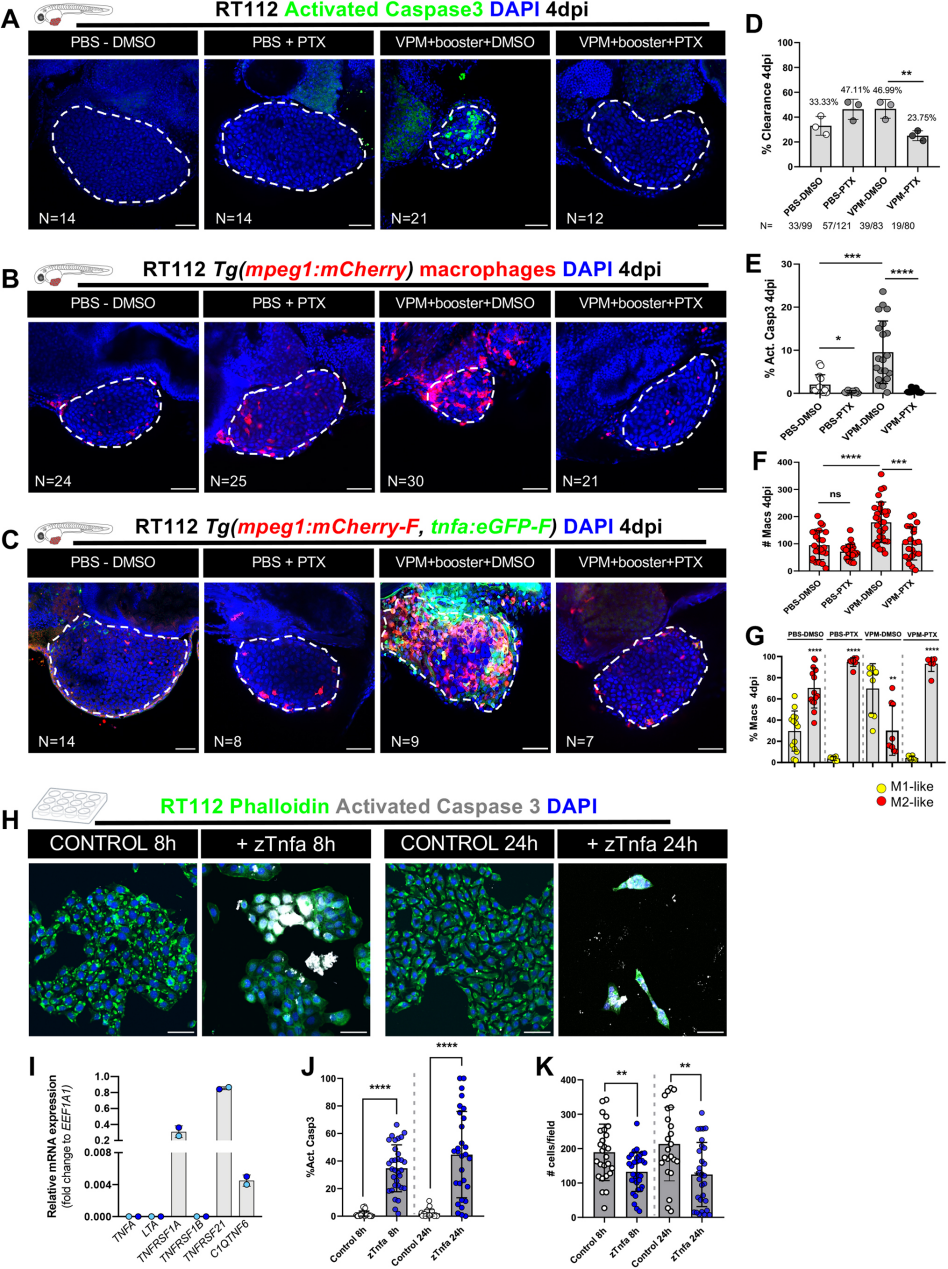
**VPM1002 induction of bladder cancer cell clearance and apoptosis depends on TNF signaling.** (A) Representative confocal images of NMIBC-RT112 control and VPM1002-treated xenografts exposed to either DMSO or pentoxifylline (PTX), in which human cancer cells were labelled with the Deep Red Cell Tracker lipophilic stain (not shown) and for the apoptosis marker activated caspase-3 (green) at 4 dpi. (B) Representative confocal images of infiltrating macrophages (red) in NMIBC-RT112 control and VPM1002+booster-treated xenografts exposed to either DMSO or PTX at 4 dpi. (C) Representative confocal images of Tnfa expression (green) and macrophages (red) in NMIBC-RT112 control and VPM1002+booster-treated xenografts exposed to either DMSO or PTX at 4 dpi. In A-C, white dashed regions outline the tumor. In all images, anterior is to the left, posterior to the right, dorsal up and ventral down. Scale bars: 50 µm. (D) Quantification of the percentage of clearance in NMIBC-RT112 control and VPM1002+booster-treated xenografts exposed to either DMSO or PTX at 4 dpi (**P=0.0031; Fisher's exact test). Each dot represents a full round of injections in which *N* represents the number of xenografts without tumors at 4 dpi relative to the total number of xenografts at 4 dpi. (E) Quantification of the percentage of activated caspase-3-positive (apoptotic) cells in NMIBC-RT112 control and VPM1002-treated xenografts exposed to either DMSO or PTX at 4 dpi (*P=0.0165; ***P=0.0002; ****P<0.0001). Each dot represents one xenograft pooled from three independent experiments. (F) Quantification of absolute numbers of infiltrating macrophages in NMIBC-RT112 control and VPM1002-treated xenografts exposed to either DMSO or PTX at 4 dpi (***P=0.0002; ****P<0.0001). Macrophages were quantified using *Tg(mpeg1:mCherry)* and *Tg(csf1ra:GFP)*. Each dot represents one xenograft pooled from three independent experiments. (G) Quantification of the percentage of M1-like (Tnfa- or NFκB-positive macrophages, Mpeg1^+^) and M2-like (Tnfa- or NFκB-negative macrophages, Mpeg1^+^) in the TME of NMIBC-RT112 control and VPM1002-treated xenografts exposed to either DMSO or PTX at 4 dpi (**P=0.0025; ****P<0.0001). Each dot represents one xenograft pooled from two independent experiments. (H) Representative confocal images of control and zebrafish Tnfa (zTnfa)-treated NMIBC-RT112 cells stained for the actin filament marker phalloidin (green), activated caspase-3 (white) and nuclei (DAPI counterstaining, blue). Scale bars: 50 µm. (I) Relative *in vitro* gene expression levels of human *TNFA*, *LTA*, *TNFRSF1A*, *TNFRSF1B*, *TNFRSF21* and *C1QTNF6* in the NMIBC-RT112 cell line. Bars indicate the fold change of expression relative to that of the housekeeping gene. Each dot represents the average of two or three technical replicates of one independent experiment. (J) Quantification of the percentage of activated caspase-3-positive cells per field in control and zTnfa-treated NMIBC-RT112 cells at 8 and 24 h post treatment. Each dot represents one random field of view (****P<0.0001). (K) Quantification of the mean absolute number of cells per field in control and zTnfa-treated NMIBC-RT112 cells at 8 and 24 h post treatment. Each dot represents one quantified well (**P<0.01). In J,K, data were pooled from two independent experiments. In D-G,I-K, bars indicate the results as mean±s.d. Data sets with a Gaussian distribution (F,G,J,K) were analyzed by parametric unpaired two-tailed *t*-test and data sets that did not pass the D'Agostino–Pearson omnibus and Shapiro–Wilk normality tests were analyzed by nonparametric unpaired Mann–Whitney test (E). Unless stated otherwise, each experimental data set was challenged to the respective control. Additionally, data sets in E-G were analyzed with Welch's one-way ANOVA with Games–Howell post hoc test in which *P*<0.0001 for the three conditions. ns, not significant, *P*≥0.05. Note that several transgenic backgrounds were used (see Table S1).

To test this, we treated xenografts with the TNF inhibitor pentoxifylline (PTX) (Marques et al., 1999) in combination with VPM1002 therapy or PBS in the controls (Fig. 8A-C). Our results show that inhibition of TNF signaling completely abrogated the clearance process (Fig. 8D), blocked the induction of apoptosis (Fig. 8E), reduced macrophage recruitment (Fig. 8F) and, as expected, also blocked the polarization of macrophages towards a pro-inflammatory phenotype (Marques et al., 1999; Kanther et al., 2011) (Fig. 8G). Note that in these experiments, we quantified M1-like and M2-like macrophages using two transgenics: *Tg(mpeg1:mCherry-F; tnfa:eGFP-F)* (Nguyen-Chi et al., 2015) and *Tg(mpeg1:mCherry; nfkb:GFP)* (Kanther et al., 2011). NFκB is a downstream target of TNF signaling and, therefore, is also used as a reporter for the TNF pathway and can be considered a marker of inflammatory macrophages (Kanther et al., 2011). Additionally, TNF inhibition abrogated the VPM1002-mediated increase of macrophages in the CHT of NMIBC-RT112 xenografts (Fig. S8), suggesting that skewing towards myelopoiesis is also mediated by TNF signaling.

These results suggest that human cancer cell killing and clearance is mediated by zebrafish-derived Tnfa. To further complement these results, we checked which TNF receptors are expressed by NMIBC-RT112 cells. Our results show that NMIBC-RT112 cells express *TNFRSF1A*, *TNFRSF21* and *C1QTNF6* (Fig. 8I). Next, we treated human NMIBC-RT112 cells with zebrafish-derived Tnfa for 8 and 24 h *in vitro* (Fig. 8H,J,K). Our results show that zebrafish-derived Tnfa led to a significant induction of apoptosis (****P<0.0001) at both timepoints of treatment and a significant decrease in the absolute number of cells per field at 8 and 24 h post exposure (**P=0.0030) (Fig. 8J,K). These results demonstrate that NMIBC-RT112 human cells are sensitive to zebrafish-derived Tnfa, further supporting the proposed mechanism of tumor clearance mediated by the BCG-induced production of zebrafish Tnfa.

## DISCUSSION

The BCG vaccine was the first successful cancer immunotherapeutic agent. BCG elicits a non-specific immune response that promotes cancer clearance and prevents recurrence (Pryor et al., 1995; Luo et al., 2003; Herr and Morales, 2008). Despite its successful history, the precise mechanisms of action of BCG, in particular, immediately after instillation, remain largely unknown (Lobo et al., 2021; Pettenati and Ingersoll, 2018; Redelman-Sidi et al., 2014; Morales et al., 1976; Prescott et al., 2000; Higuchi et al., 2009). In this work, we set to elucidate the initial anti-tumoral mechanisms of action of BCG through the use of the zebrafish bladder cancer xenograft model. For this, we focused on the crosstalk between BCG and innate immunity, which initiates the cascade of responses to therapy.

We showed *in vivo* that BCG induced tumor clearance and apoptosis of human bladder cancer cells and that this effect was mediated by macrophages. Immediately after BCG treatment, macrophages massively infiltrate tumors and become polarized towards a pro-inflammatory phenotype (M1-like, Tnfa positive), accompanied by an induction of several inflammatory cytokines such as *tnfa*, *il1b* and *il6.* Depletion of macrophages with L-clodronate completely abrogated the BCG anti-tumor effects, demonstrating that clearance and apoptosis are dependent on macrophage activity. Long-term light-sheet microscopy revealed that macrophages altered their behavior in response to BCG, increasing phagocytosis, macrophage cell–cell interactions and macrophage fusion events. Next, we showed that cancer cell clearance highly depends on TNF signaling. Importantly, expression of several key myelopoietic progenitor transcription factors was increased in the CHT upon BCG or VPM1002 treatment, suggesting skewing of the hematopoietic compartment towards myelopoiesis. Moreover, we provide proof-of-concept experiments demonstrating that our model was able to discern distinctive innate immune responses to two different BCG vaccine strains – the conventional BCG and the recombinant second-generation BCG-based vaccine VPM1002.

These findings provide key insights into the initial processes involved in BCG immunotherapy. We challenge the notion that macrophages are only antigen-presenting cells and secrete cytokines to induce an effective adaptive response. We show that, in contrast to what is shown in the current BCG-induced tumor immunity model (Pettenati and Ingersoll, 2018), macrophages are also able to directly induce apoptosis and clear cancer cells *in vivo*. This is in accordance with a previous report that indicates that macrophages can have direct anti-tumor activity *in vitro* (Luo et al., 2010). In this work, the authors show that macrophages and T lymphocytes can directly kill bladder cancer cells upon BCG stimulation, with T lymphocytes having a higher anti-tumoral activity. So far, we could not find any *in vivo* reports showing this direct active role of macrophages.

In all, our work suggests a new step to the multi-step model of BCG-induced tumor immunity right after the initial step: an earlier stage in which macrophages are able to directly kill and clear tumor cells. Nevertheless, some cancer cells still escape (shown in our model by the few tumors that remained uncleared after BCG treatment). Then, macrophages that are no longer able to kill and clear tumor cells call forth the adaptive immune response through the expression of cytokines, chemokines and antigen presentation, fully inducing a complete immune response to clear the remaining tumor cells.

Macrophages are innate immune cells with unique transcriptional diversity and the capacity to switch their phenotype and function in response to diverse stimuli. Additionally, macrophages are crucial in the development of pathologies caused by different members of the genus *Mycobacterium* (including BCG) (Upadhyay et al., 2018), such as TB and leprosy (Madigan et al., 2017a,b; Roca et al., 2019; Osman et al., 2022). Therefore, we focused on a deeper understanding of the role of macrophages in the anti-tumoral effects of the BCG vaccine. Several studies have shown that the bladder cancer TME is highly immunosuppressive (Wang et al., 2017; Martínez et al., 2017), with anti-inflammatory macrophages (M2-like) being the main cellular subset found in histopathological samples from patients with BCG treatment failure or BCG resistance (Takayama et al., 2009; Takeuchi et al., 2016; Suriano et al., 2013). In accordance, we also observed that untreated bladder cancer xenografts had a TME enriched in anti-inflammatory (M2-like Tnfa negative) macrophages. However, upon BCG treatment, there was an induction of an inflammatory TME together with tumor clearance and apoptosis, which was highly dependent on TNF signaling.

We reveal that the presence of the BCG vaccine in the TME was sufficient to immediately trigger a brisk change in macrophage dynamics. Macrophages were highly mobile in response to two different vaccine strains, the conventional BCG and VPM1002. However, those exposed to VPM1002 were more inflammatory and efficient at inducing tumor apoptosis. These results highlight the notion that not all immune cell infiltrates are similar and that further features should be analyzed to predict treatment response.

Despite the fact that we did not observe any differences in neutrophil infiltration at 4 dpi, we do not discard the possibility of changes in neutrophil phenotypes upon BCG treatment at earlier or later timepoints in our assay.

Interestingly, we found that in the absence of cancer cells, although BCG or VPM vaccines induced an inflammatory cytokine response (induction of *tnfa*, *il1b* and *il6*), this was not translated into a marked innate cellular response. In line with this, in healthy human volunteers, intradermal BCG vaccination does prompt a mild systemic inflammatory response but not cellular changes (Cirovic et al., 2020; Moorlag et al., 2020). In contrast, the presence of human cancer cells alone induced a clear cellular response. However, when cancer cells were combined with the vaccines, this induced a much stronger inflammatory response, which also translated into a more robust cellular response.

Live-imaging analysis showed that macrophages acquired different phenotypes in response to BCG. From the different phenotypes displayed, we identified fusion events among phagocytic macrophages in the xenografts that were treated with BCG. Fusion events were more prevalent in the VPM1002-treated xenografts. Here, phagocytic macrophages came in close contact and appeared to fuse with each other. These macrophages resembled granulomatous multinucleated giant cells (MGCs). MGC formation is a macrophage-specific event that is highly evolutionarily conserved (Pagán and Ramakrishnan, 2018). Although MGC function is not clearly defined, it has been proposed that this event promotes more profound phagocytic and antimicrobial capacities (Pagán and Ramakrishnan, 2018). Thus, we speculate that the macrophage fusion observed in long-term imaging experiments was the beginning of the formation of MGCs in early granuloma, supporting previous studies that revealed that granuloma formation is an earlier event than as previously shown (Davis and Ramakrishnan, 2009; Saunders and Cooper, 2000).

Upon inhibition of TNF signaling, VPM1002 failed to induce tumor clearance and apoptosis. TNF is required for host protection against mycobacterial infections and for granuloma formation (Kindler et al., 1989; Chavez-Galan et al., 2019). TNFA is a transmembrane protein that mediates cell–cell contact-dependent apoptosis. This process is achieved through the binding of TNFA to its receptor TNFRSF1A, which is generally highly expressed in cancer cells (Boyle et al., 2003; Josephs et al., 2018; Declercq et al., 1998). NMIBC-RT112 cells express three different TNF receptors TNFRSF1A, TNFRSF21 and C1QTNF6. We show that the zebrafish Tnfa protein can induce NMIBC-RT112 apoptosis *in vitro*, demonstrating conservation and crosstalk between zebrafish Tnfa and human TNF receptors. It has been previously shown that similar to human TNFA, zebrafish Tnfa forms trimers and, thus, it is possible that zebrafish Tnfa and human TNFRSF1A bind (Duan et al., 2021). As NMIBC-RT112 cells express TNFRSF1A, we speculate that this might indeed be the human receptor that is binding zebrafish Tnfa.

We speculate that BCG-induced contact-dependent macrophage killing also takes place in patients with cancer, as the abundance of TNF in the urine of patients with bladder cancer is markedly increased after BCG instillation (Bisiaux et al., 2009). Consistently, macrophages of patients with gastric cancer that received BCG immunotherapy expressed high levels of TNFA (Zembala et al., 1993).

Novel therapeutic approaches focused on the adaptive immune system are among the leading therapies for BCG resistance in patients with bladder cancer (Witjes et al., 2021). Unfortunately, when used as single agents, these therapies only benefit a small number of patients and have numerous adverse events (Sharma and Allison, 2015). It has been suggested that several of these therapies fail due to the presence of immunosuppressive innate immune cells, predominantly macrophages and monocytes (Smith and Zaharoff, 2016; Joseph and Enting, 2019). Along this line, patients with bladder cancer treated with aspirin, an inhibitor of cyclooxygenase (COX) 1 and 2 (encoded by *PTGS1* and *PTGS2*, respectively), while undergoing intravesical immunotherapy benefited from better response rates (Lipsky et al., 2013). In keeping with these results, it was previously shown that COX-2-driven inflammation stimulates the infiltration of immunosuppressive myeloid cells to the TME, which, in turn, impairs responses to checkpoint inhibitors (Zelenay et al., 2015). Thus, modulating the innate immune system, in particular, macrophages, will likely boost the anti-tumor effects of checkpoint inhibition (Netea et al., 2017).

Our findings show that the zebrafish xenograft model has the potential to provide a real-time window with single-cell resolution to test and mechanistically understand new therapies targeting the innate immune system, in particular, innate immunomodulatory drugs or vaccines. These new drugs and vaccines could be then combined with immune checkpoint therapies to engage both arms of the immune system in the fight against cancer.

## MATERIALS AND METHODS

### Zebrafish husbandry

Zebrafish (*Danio rerio*) were handled and maintained according to the standard protocols of the European Animal Welfare Legislation, Directive 2010/63/EU (European Commission, 2016), and the Champalimaud Foundation Fish Platform. All protocols were approved by the Champalimaud Animal Ethical Committee and Portuguese institutional organizations – Órgão de Bem-Estar e Ética Animal (ORGEA; Animal Welfare and Ethics Body) and Direção Geral de Alimentação e Veterinária (DGAV; Directorate General for Food and Veterinary).

Zebrafish, between 3 and 18 months of age, were reared in 3.5 l tanks at a density of 10 fish/l with females and males together. The rearing temperature was 28°C. Animals were kept in a light/dark cycle of 14 h/10 h (lights on from 08:00 until 22:00). Zebrafish were fed three times per day, artemia in the mornings, and powder (Sparos 400-600, U000001864, Techniplast) in the afternoons and evenings.

### Zebrafish transgenic lines

According to the purpose of each experiment, different genetically modified zebrafish lines were used in this study: *Tg*(*mpx:GFP*) i114Tg (Renshaw et al., 2006), *Tg(mpeg1:mCherry*) ump2 (Ellett et al., 2011), *Tg(csf1ra:GFP)* sh377Tg (Dee et al., 2016), *Tg(mpeg1:mCherry-F; tnfa:GFP-F*) ump2Tg;ump5Tg (Nguyen-Chi et al., 2015), *Tg(mpeg1:mCherry; nfkb:GFP)* ump2Tg;nc1Tg (Kanther et al., 2011), *Tg(fli:GFP)* y1Tg (Lawson and Weinstein, 2002) and the *mitfa^b692^* (*nacre*) line (Lister et al., 2001). In one experiment, an outcross of *Tg(mpx:GFP)* and *Tg(mpeg1:mCherry)* was performed to obtain double-transgenic animals to simultaneously quantify neutrophils and macrophages (Fig. 7). All the zebrafish transgenic lines and *mitfa^b692^* (*nacre*) fish are in the Tübingen background. Most adults of each transgenic line are *nacre*^−/−^, but some are *nacre*^+/−^ to maintain genetic variability; thus, some embryos might have pigmentation, and we also used them in the experiments. Several experiments were performed in parallel and in several transgenic backgrounds in order to use the same controls, therefore reducing the number of animals (as per the principles of the 3Rs – Replacement, Reduction and Refinement). In the legends, we indicate which experiments were performed in parallel and therefore share controls. See Table S1 for information on which lines were used in each experiment and figure.

### Human cancer cell lines and culture

Human urothelial cancer RT112 (female) and J82 (male) cell lines were a kind gift from Dr Mireia Castillo (Champalimaud Foundation, Portugal). Cell lines were authenticated by small tandem repeat profiling using ‘fast technology for analysis of nucleic acids’ (FTA) cards (STAB vida, Portugal) and were routinely mycoplasma tested. Both cell lines were kept and grown in Dulbecco's modified Eagle medium (DMEM) High Glucose (L0102, Biowest) and supplemented with 10% fetal bovine serum (FBS; Sigma-Aldrich) and antibiotics (100 U/ml penicillin and 100 µg/ml streptomycin, Hyclone) in a humidified 5% CO_2_ atmosphere at 37°C.

### Cell staining

Tumor cells were grown to 85-90% confluence in T-175 flasks, washed with 1× Dulbecco's phosphate-buffered saline (DPBS) (Biowest) and detached enzymatically using TrypLE (Thermo Fisher Scientific). Cell suspensions were collected in 15 ml centrifuge tubes, spun down at 300* **g*** for 4 mins and resuspended in 1× DPBS. Cells were then stained in 1.5 ml microcentrifuge tubes using lipophilic dyes – Vybrant CM-DiI (4 µl/ml in 1× DPBS) or Deep Red Cell Tracker (1 µl/ml in 1× DPBS, 10 mM stock) (Life Technologies) – for 15 min at 37°C while protected from light. Cells were washed by spinning down at 300* **g*** for 5 min at 4°C and resuspended in complete medium. Viability was assessed by the Trypan Blue exclusion method, and cell number was determined by hemocytometer counting. Cells were resuspended in complete medium to a final concentration of 0.5×10^6^ cells/µl.

### Bacterial strains

The recombinant BCG*ΔureC::hly* (VPM1002) (Grode, 2005; Rentsch et al., 2022), BCG:SSI pGFP (Grode, 2005; Rentsch et al., 2022) and BCG:SSI pmCherry (Grode, 2005; Rentsch et al., 2022) were provided by the Department of Immunology, Max-Planck Institute for Infection Biology (MPIIB), Germany. OncoTICE^®^ (BCG Strain TICE^®^, Merck) was provided by the Urology Unit, Champalimaud Foundation.

### Bacterial culture

Glycerol-frozen bacteria were thawed on ice for ∼3-4 h. Thawed bacteria were spun down at 3000* **g*** for 10 min and washed twice in 1× PBS. Pelleted bacteria were resuspended in 100 µl of 1× PBS, seeded on Middlebrook 7H11 agar plates supplemented with 10% OADC (M0678, Sigma-Aldrich) and incubated at 37°C until colony formation (∼4-5 weeks). Fluorescent BCG:SSI colonies were selected and grown in 5 ml of liquid Middlebrook 7H9 broth supplemented with 10% ADC (M0553, Sigma-Aldrich) and 50 µg/ml hygromycin (H7772, Sigma-Aldrich) in 50 ml centrifuge tubes at 37°C, with shaking at 50 rpm until high turbidity was reached. 1 ml aliquots of highly concentrated bacterial cultures were seeded into 9 ml of Middlebrook 7H9 broth containing 10% ADC and 50 µg/ml hygromycin in 30 ml sterile bottles (2019-0030, Thermo Fisher Scientific) and incubated at 37°C, with shaking at 50 rpm until the cultures reached an optical density at 600 nm (OD_600_) of 0.8.

VPM1002 colonies were selected and grown in 5 ml of liquid Middlebrook 7H9 broth supplemented with 10% ADC in 50 ml centrifuge tubes at 37°C, with shaking at 50 rpm until high turbidity was reached. 1 ml aliquots of highly concentrated bacterial cultures were seeded into 9 ml of Middlebrook 7H9 broth containing 10% ADC in 30 ml sterile bottles and incubated at 37°C, with shaking at 50 rpm until the cultures reached an OD_600_ of 1.2.

Once the desired optical density was reached, bacteria were spun down at 3000* **g*** for 10 min. Pelleted bacteria were then washed and resuspended in 1× PBS, from which a sample was streaked in Middlebrook 7H11 plates for enumeration of colony-forming units (CFUs). Bacteria were spun down again and resuspended in 10% glycerol in PBS solution, frozen in cryovials and stored at −80°C. In order to check for contamination, an aliquot of bacterial culture was streaked on LB agar plates at different timepoints of the protocol and incubated at 37°C.

OncoTICE^®^ vials were resuspended in sterile 0.9% sodium chloride solution at the Day Hospital (Champalimaud Foundation) according to the manufacturer instructions (Merck, one vial/50 ml saline solution). Remnants from the resuspended vials were stored at 4°C and protected from light.

### Bacterial staining

OncoTICE^®^ vials were spun down at 3000* **g*** for 10 min, the supernatant was carefully discarded and pelleted bacteria were resuspended in lipophilic dye solutions – Vybrant CM-DiI (4 µl/ml in 1× PBS) or Deep Red Cell Tracker (1 µl/ml in 1× PBS, 10 mM stock). Bacteria were then incubated at 37°C with shaking at 300 rpm for 30 min while protected from light. Labelled bacteria were spun down at 3000* **g*** for 5 min, washed once with 1× PBS and resuspended to the desired concentration in 1× PBS.

### *In vitro* challenge with BCG

RT112 and J82 cells were seeded in 24-well plates previously lined with sterile coverslips and incubated in a humidified 5% CO_2_ atmosphere at 37°C. Both cell lines were challenged on days 1 and 3 after seeding with either 1× DPBS (control) or 10× BCG [OncoTICE^®^, (1-8)×10^8^ CFUs]. On day 4 after seeding, the cell medium was removed, cells were washed and fixed in 4% (v/v) formaldehyde (FA) for 10 min and immunofluorescence staining was immediately performed.

### *In vitro* challenge with zebrafish Tnfa

At 80% confluence, NMIBC-RT112 cells were plated onto coverslips into a 12-well plate (Corning) at approximately 0.075×10^6^ cells per well and incubated in growth medium. Upon reaching 70-80% confluence, cells were treated with 100 ng/ml recombinant zebrafish Tnfa (RP1318Z-005, Kingfisher Biotech) or left untreated (control, PBS with 0.1% bovine serum albumin as vehicle) for 8 and 24 h, after which cells were fixed in 4% (v/v) FA for 10 min at room temperature (RT).

### Immunofluorescence staining for *in vitro* cultures

FA-fixed cells were washed twice for 5 min with 500 µl of 1× PBS at RT. Cells were permeabilized by incubation at RT with 0.1% Triton X-100 in 1× PBS for 25 min. Cells were blocked in 500 µl of PBDX_GS (50 ml of 1× PBS, 0.5 g bovine serum albumin, 0.5 ml DMSO, 0.25 ml of 1% Triton X-100 and 0.75 ml goat serum) for 1 h at RT. Cells were stained with Alexa Fluor 488 Phalloidin (A12379, Thermo Fisher Scientific, 1:200) and rabbit anti-cleaved caspase-3 (Asp175) (9661, Cell Signaling Technology, 1:250) inside a humid chamber at 4°C overnight. The next day, cells were washed three times with 1× PBS for 5 min at RT. Cells were then incubated in 30 µl of diluted secondary antibody (84546, Thermo Fisher Scientific, 1:500 in PBDX_GS) on top of a sheet of parafilm, inside a humid chamber at 4°C overnight and protected from light. After incubation, cells were washed twice in 1× PBS for 5 min at RT. DNA was counterstained with DAPI for 10 min at a concentration of 50 µg/ml in PBS while protected from light. Cells were then washed twice in distilled water for 5 min at RT. Coverslips were then dried and mounted on microscope glass slides using Mowiol aqueous mounting medium (81381, Sigma-Aldrich). Slides were stored at 4°C protected from light.

### Zebrafish xenografts

On the injection day, hatched embryos were separated from unhatched eggs. 1× pronase (10165913103, Roche) was added to the embryo medium to boost hatching. The embryos were anesthetized by incubation in 1× tricaine for 5 min. Approximately 50 anesthetized embryos were transferred to an agar/agarose plate. The embryos were carefully aligned in the agar/agarose plate with the help of a hairpin loop. Fluorescently labelled cancer cells were injected using a microinjection needle under a stereomicroscope (ZEISS Stemi 305) with a milli-pulse pressure injector (Applied Scientific Instrumentation, MPPI-3). The treated embryos were transferred to a clean standard Petri dish with 1× tricaine solution and left to rest for 10 min to allow the wound to close. Treated embryos were then placed in fresh E3 medium and incubated at 34°C. At 1 dpi, zebrafish xenografts were screened for the presence or absence of tumoral masses in a fluorescence stereomicroscope (Zeiss Axio Zoom V16). Xenografts with edema, cells in the yolk sac and cellular debris were discarded. At 4 dpi, zebrafish xenografts were analyzed using the fluorescence stereomicroscope and the clearance rate was quantified as follows:

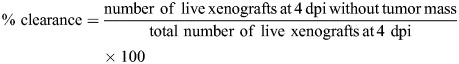


### Zebrafish macrophage ablation with clodronate liposomes

At 1 dpi and 3 dpi, xenografts were anesthetized by incubation in 1× tricaine for 5 min. For the selective ablation of macrophages, ∼14 nl of either liposome-encapsulated PBS (L-PBS) or liposome-encapsulated clodronate (L-clodronate) (CP-005-005, Liposoma, 5 mg/ml) were injected intratumorally at a 0.5× concentration using a microinjection needle under a stereomicroscope with a milli-pulse pressure injector. Treated xenografts were placed immediately in clean E3 medium and incubated at 34°C.

### Chemotherapy of zebrafish xenografts

At 1 dpi, zebrafish were randomly distributed into control and treatment groups. The maximum tolerated concentration of drugs in zebrafish larvae was determined as previously described (Fior et al., 2017). Zebrafish were then anesthetized by incubation in 1× tricaine for 5 min and ∼14 nl of L-PBS, L-clodronate, mitomycin C (0.5 mg/ml; Medac) with L-PBS or mitomycin C with L-clodronate was injected intratumorally, and xenografts were placed immediately in clean E3 medium. This procedure was repeated at 3 dpi. Throughout the experiment, xenografts were kept at 34°C and assessed daily. Xenografts were euthanized and fixed at 4 dpi in 4% (v/v) FA overnight at 4°C, and transferred the next day to 100% (v/v) methanol at −20°C.

### BCG immunotherapy of zebrafish xenografts

At 1 dpi, zebrafish were randomly distributed into control and treatment groups. BCG stock vials were thawed on ice, spun down at 3000 ***g*** for 10 min and washed twice in 1× PBS. Bacteria were passed through a 25G needle to promote single cell dilution and resuspended in 1× PBS to a final concentration of 3-4×10^6^ CFU/ml. Xenografts were anesthetized with 1× tricaine. ∼14 nl of L-PBS, L-clodronate, BCG or BCG with L-clodronate was injected intratumorally and xenografts were placed immediately in clean E3 medium. This procedure was repeated at 3 dpi. Throughout the experiment, xenografts were kept at 34°C and assessed daily. Xenografts were euthanized and fixed at 4 dpi in 4% (v/v) FA overnight at 4°C, and transferred the next day to 100% (v/v) methanol at −20°C.

### Single-cell light-sheet live imaging and analysis of zebrafish xenografts

At 1 dpi, control, BCG-treated or VPM1002-treated *Tg(csf1ra:GFP)^sh377^* xenografts were left to rest in E3 medium for ∼5 min immediately after treatment. A single xenograft was then chosen and mounted in a capillary tube with 0.8% low-melting agarose. The mounted xenograft was placed inside the chamber of a Zeiss Light Sheet Z.1 microscope, previously filled with 0.75× tricaine in E3 medium without Methylene Blue at 34°C. Using a 20× objective lens and the Zen Blue software, the area of the tumor was delimited and *z*-stack images were acquired every 3 min with a 5 µm interval between slices. Xenografts were imaged for ∼15 h and then euthanized.

Light-sheet files were converted to HDF5/XML files using the BigDataViewer plugin from ImageJ/Fiji Software (Pietzsch et al., 2015). Randomly selected individual macrophages were manually tracked in three dimensions using the MaMut plugin from ImageJ/Fiji (Wolff et al., 2018). Motion analysis (maximum distance traveled, total distance traveled and mean speed) was based on the TrackMate algorithms in ImageJ/Fiji (Ershov et al., 2022).

For the quantification of elongated macrophages, phagocytosis, macrophage touching and macrophage fusion, three maximum-intensity projections (MIPs) of each tumor were assessed. Tumors were divided in thirds in relation to their *z*-plane and a MIP was created from each third. Then, each event was manually quantified along the 15 h of imaging per MIP (∼900 images per tumor). Data were exported as CSV files and statistical analysis was performed using GraphPad Prism 8.0 software.

### Immunofluorescence

Xenografts stored in 100% methanol were rehydrated by a series of decreasing methanol concentrations (75%, 50% and 25% methanol with 0.1% Triton X-100 in 1× PBS). Xenografts were washed four times for 5 min in 0.1% Triton X-100 in 1× PBS, then washed once for 5 min in MilliQ H_2_O. Next, xenografts were incubated on ice-cold acetone at −20°C for 7 min and washed twice for 10 min in 0.1% Triton X-100 in 1× PBS. Then, they were incubated at RT for 1 h in PBDX_GS blocking buffer. PBDX_GS was removed and ∼40 µl of diluted primary antibody was added [rabbit anti-cleaved caspase-3 (Asp175), 9661, Cell Signaling Technology, 1:100 in PBDX_GS]. Xenografts were incubated at RT for 1 h and then overnight at 4°C. The primary antibody was removed and xenografts were washed twice for 10 min in 0.1% Triton X-100 in 1× PBS. Then, they were washed four times for 30 min in 0.05% Tween 20 in 1× PBS. The 0.05% Tween 20 in 1× PBS was removed and ∼40 µl of diluted secondary antibody (A11034, Alexa Fluor 488, Life Technologies; Dylight 594, 35560, Thermo Fisher Scientific; Dylight 650, 84546, Thermo Fisher Scientific; 1:200 in PBDX_GS) with DAPI (50 µg/ml) was added. Xenografts were incubated at RT for 1 h and then overnight at 4°C. The diluted secondary antibody was removed and xenografts were washed four times for 15 min in 0.05% Tween 20 in 1× PBS. Xenografts were fixed in 4% FA for 20 min and washed once in 0.05% Tween 20 in 1× PBS for 10 min. Xenografts were then mounted in Mowiol aqueous mounting medium between two coverslips to allow for double-side microscope acquisition.

### Confocal imaging and analysis of zebrafish xenografts

Mounted xenografts were imaged using an inverted LSM 710 confocal microscope (Zeiss) with Zen software or an Andor BC43 spinning disk confocal microscope. Tumors were imaged with a 25× immersion objective lens or a 20× objective lens using the *z*-stack function with an interval of 5 µm between slices. The number of cells was manually assessed with the Cell Counter plugin from ImageJ/Fiji. To assess tumor size, a proxy of the total cell number (DAPI nuclei) was estimated by counting the number of nuclei in three representative slices of the tumor from the top (Z_first_), middle (Z_middle_) and bottom (Z_last_) per *z*-stack per xenograft, as follows:

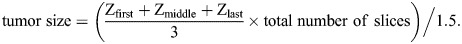


The 1.5 correction number was estimated for human cells that have a nucleus with an average diameter of 10-12 µm. The numbers of activated caspase-3-positive cells, macrophages, neutrophils, Tnfa-positive/negative macrophages was individually quantified in every slice along the tumor. To get the percentage of each population, the obtained number was divided by its corresponding tumor size.

Whole-body images of zebrafish larvae were obtained by tiling of images (ImageJ Pairwise Stitching plugin) or by automated tiling in the Andor BC43 spinning disk Fusion software.

### Histopathology

Fish were euthanized, fixed in 4% FA and longitudinally embedded in paraffin. 4 µm serial sections were cut using a HM340E microtome (Thermo Fisher Scientific) and stained with Hematoxylin and Eosin or Ziehl-Neelsen color kit (80276, Liofilchem). Tissue sections were examined by a pathologist from the Champalimaud Foundation Histopathology platform using an Axioscope 5 microscope (Zeiss) and microphotographs captured with an Axiocam 208 color camera (Zeiss).

### Whole-mount *in situ* hybridization

Zebrafish xenografts at 4 dpi were collected and fixed in 4% FA at 4°C overnight, dehydrated through a methanol series and stored in 100% methanol at −20°C. Whole-mount *in situ* hybridization was performed as described (Thisse et al., 1993) with minor modifications (hybridization temperature, 65°C), using digoxigenin-labelled antisense RNA probes for *lcp1* and *c-myb* (a gift from the Rui Monteiro laboratory, Institute of Cancer and Genomic Sciences, University of Birmingham, UK). The staining reaction was performed using BMP-Purple (Roche). Zebrafish larva xenografts were photographed using a Zeiss SteREO Discovery.V8 stereomicroscope coupled to a Zeiss AxioCam Icc 3 Camera.

### RNA isolation, reverse transcription and RT-qPCR

Between 100 and 200 RT112-injected xenografts in *Tg(mpx:GFP)* and *Tg(mpeg1:mCherry)* at 4 dpi were dissected into the head, trunk and tail. The different tissue regions were collected into a mixture of DMEM High Glucose supplemented with 10 µM of anoikis inhibitor Y-27632 (S1049, Selleckchem) and 5 U DNAse I (RNase-free) (EN0521, Thermo Fisher Scientific), and kept on ice during the procedure. Tissues were centrifuged for 300 ***g*** for 5 min 4°C. The supernatant was discarded and the tissues were resuspended in TRIzol (15596026, Thermo Fisher Scientific) until they were fully dissociated. RNA was isolated with a combination of phenol-chloroform (P3803, Sigma-Aldrich) and RNeasy Mini Kit (74104, Qiagen), and cDNA was generated with the Xpert cDNA synthesis kit (GK80.0100, GRiSP) according to the manufacturer's instructions in a C1000 Touch Thermal Cycler (Bio-Rad). For RT-qPCR, 100 ng of template cDNA was used per reaction and primers were used at 0.4 µM. SYBR Blue Mastermix (GE22.5100, GRiSP) was used according to the manufacturer's instructions, and the reactions were performed in a CFX96 Real-Time System C1000 Touch Thermal Cycler (Bio-Rad).

Human and zebrafish primers were synthetized by Integrated DNA Technologies and are listed in Table S2. Gene expression levels were normalized to the housekeeping genes *EEF1A1* (human) or *eef1a1a* (zebrafish). Relative mRNA expression levels were calculated using the following formulas: (1) fold change relative to housekeeping gene expression=2^−ΔCt^, where ΔCt=cycle threshold value (Ct) of the gene of interest – Ct of the housekeeping gene; and (2) fold change relative to expression in the control condition=2^−ΔΔCt^, where ΔΔCt=ΔCt for the experimental condition – ΔCt for the control condition.

### Statistical analysis

Statistical analysis was performed using the GraphPad Prism 8.0 software. All data sets were challenged by D'Agostino and Pearson and Shapiro–Wilk normality tests. In general, data sets with a Gaussian distribution were analyzed by parametric unpaired two-tailed *t*-test and data sets that did not pass the normality tests were analyzed by nonparametric unpaired Mann–Whitney test. Unless stated otherwise, each experimental data set was challenged to the respective control. Clearance data sets were analyzed using Fisher's exact test. All were two-sided tests with a confidence interval of 95%. Differences were considered significant at *P*<0.05 and statistical output was represented as follows: ns, not significant, *P*≥0.05; *P<0.05; **P<0.01; ***P<0.001; ****P<0.0001. Bars indicate the results as mean±standard deviation of the mean (s.d.). When comparing more than two conditions, Welch's one-way ANOVA with Games–Howell post hoc test was also applied (normal distribution).

## Supplementary Material

10.1242/dmm.050693_sup1 Supplementary information
