# Supplementary material for: Macrophages directly kill bladder cancer cells through TNF signaling as an early response to BCG therapy
Source: Dis Model Mech. 2024 Aug 8;17(8):dmm050693. doi: 10.1242/dmm.050693 (PMC11554267; doi:10.1242/dmm.050693)
Supplement: Supplementary information [file dmm-17-050693-s1.pdf]

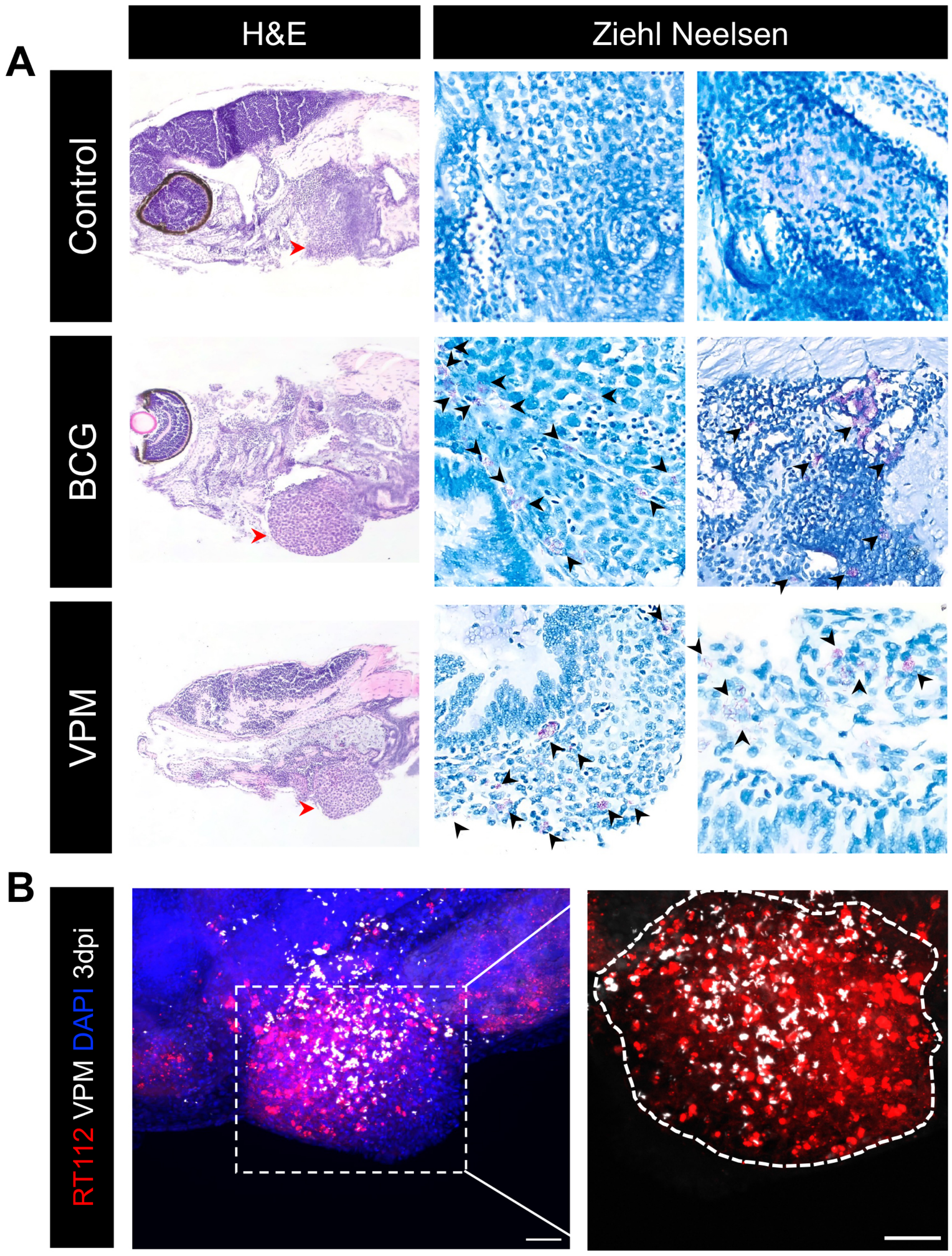

Fig. S1. A zebrafish xenograft model for BCG immunotherapy in bladder cancer. A) Representative microphotographs of zebrafish xenografts, stained with Hematoxylin and Eosin (first column, red arrow heads point to the tumor) and with Ziehl Neelsen (second and third column) 24h after bacteria injection. Acid-fast bacilli, staining bright red with Ziehl Neelsen (black arrow heads) are seen within some of the tumors, inside macrophages, extracellularly and, more rarely, inside tumor cells. **B)** Representative confocal images of NMIBC-RT112 VPM1002-treated xenografts with human cancer cells labelled with the Vybrant CM-DiI lipophilic staining (red) and VPM1002 labelled with the Deep Red Cell staining (white) 1h after boost injection. Scale bar: 50µm.

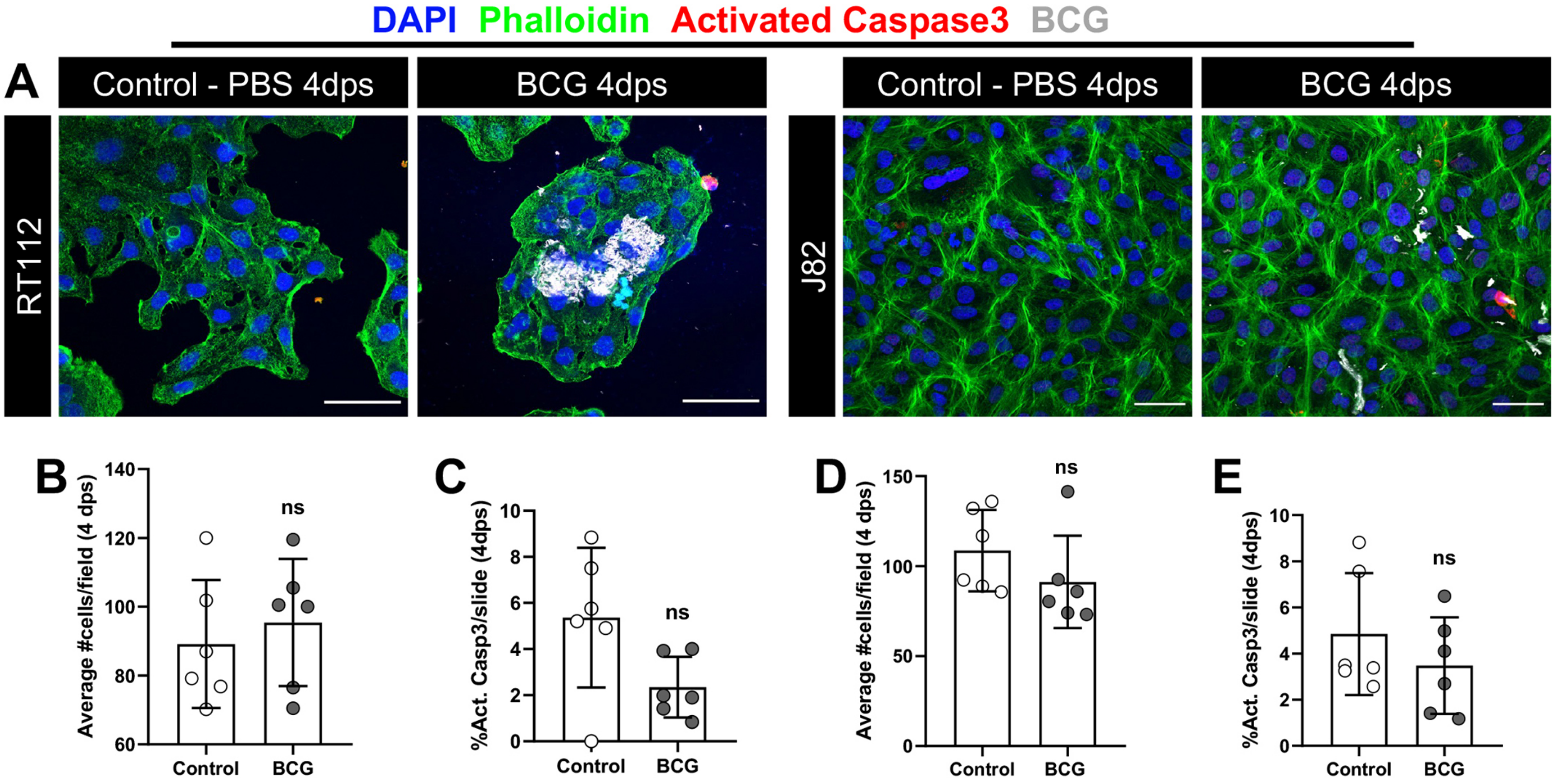

Fig. S2. NMIBC-RT112 and MIBC-J82 cell lines are not susceptible to BCG *in vitro*. A) Representative confocal images of NMIBC-RT112 and MIBC-J82 cells stained for the actin filaments marker phalloidin (green), apoptosis marker activated caspase 3 (red), BCG (white) and DAPI nuclei counterstaining. **B)** Quantification of the mean absolute number of cells per field in control and BCG-treated NMIBC-RT112 cells at 4dps. **C)** Quantification of the percentage of activated caspase 3 cells per field in control and BCG-treated NMIBC-RT112 cells at 4dps. **D)** Quantification of the mean absolute number of cells per field in control and BCG-treated MIBC-J82 cells at 4dps. **E)** Quantification of the percentage of activated caspase 3 cells per field in control and BCG-treated MIBC-J82 cells at 4dps. Bars indicate the results as AVG ±STDEV and each dot represents one quantified well. Data pooled from 2 independent experiments. Average cells/field and activated caspase 3 expression data sets were analyzed by a parametric unpaired t-test. Differences were considered significant at $P < 0.05$ and statistical output was represented as follows: non-significant (ns) ≥0.05, *<0.05, **<0.01, ***<0.001, ****<0.0001. Scale bar: 50μm. dps: days post-seeding.

Disease Models & Mechanisms • Supplementary information

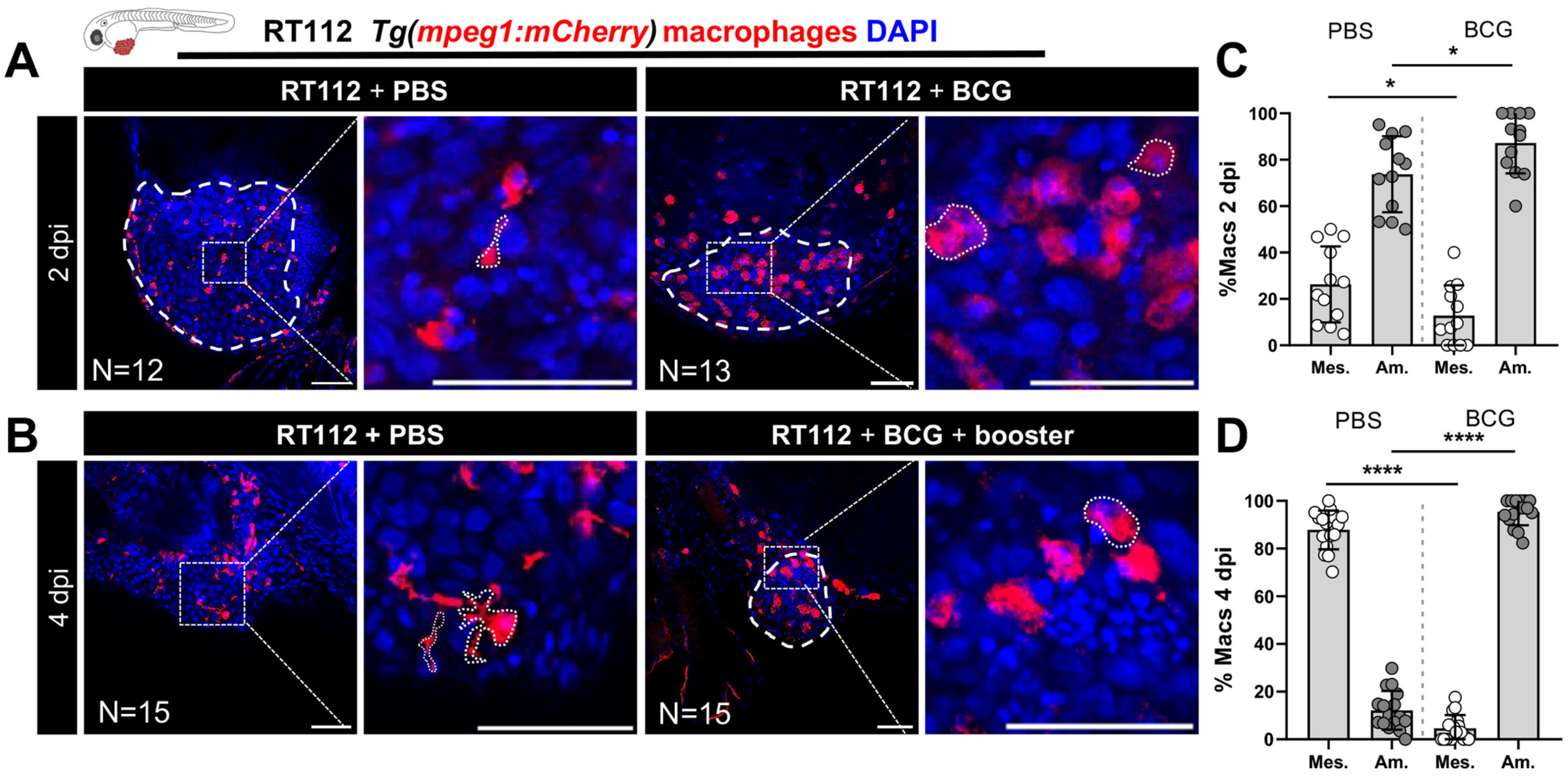

Fig. S3. BCG treated xenografts comprise more macrophages with ameboidal morphology. A and **B)** Representative confocal images of infiltrating macrophages (red) in NMIBC- RT112 control and BCG-treated xenografts at 2 and 4dpi in which human cancer cells were labelled with the Deep Red Cell Tracker lipophilic staining. Big white dotted lines outline the tumor and small dotted lines outline macrophages with either mesenchymal or round/ameboidal morphology. **C** and **D)** Quantification of the percentage of infiltrating macrophages with either a mesenchymal or ameboidal morphology in NMIBC-RT112 control and BCG-treated xenografts at 2dpi (mesenchymal *P=0.0370, ameboidal *P=0.0370) and 4dpi (****P<0.0001). Bars indicate the results as AVG ± STDEV and each dot represents one xenograft pooled from 2 independent experiments. All data sets were challenged by D'Agostino & Pearson and Shapiro–Wilk normality tests. Data sets with a Gaussian distribution were analyzed by Welch's parametric unpaired t test and data sets that did not pass the normality tests were analyzed by nonparametric unpaired Mann–Whitney test. Unless stated otherwise, each experimental dataset was challenged to the respective control. All were two-sided tests with a confidence interval of 95%. Differences were considered significant at P < 0.05 and statistical output was represented as follows: non-significant (ns) ≥0.05, *<0.05, **<0.01, ***<0.001, ****<0.0001. All images are anterior to the left, posterior to right, dorsal up and ventral down. Scale bar: 50 µm. dpi: days post-injection. TME: tumor microenvironment.

Disease Models & Mechanisms • Supplementary information

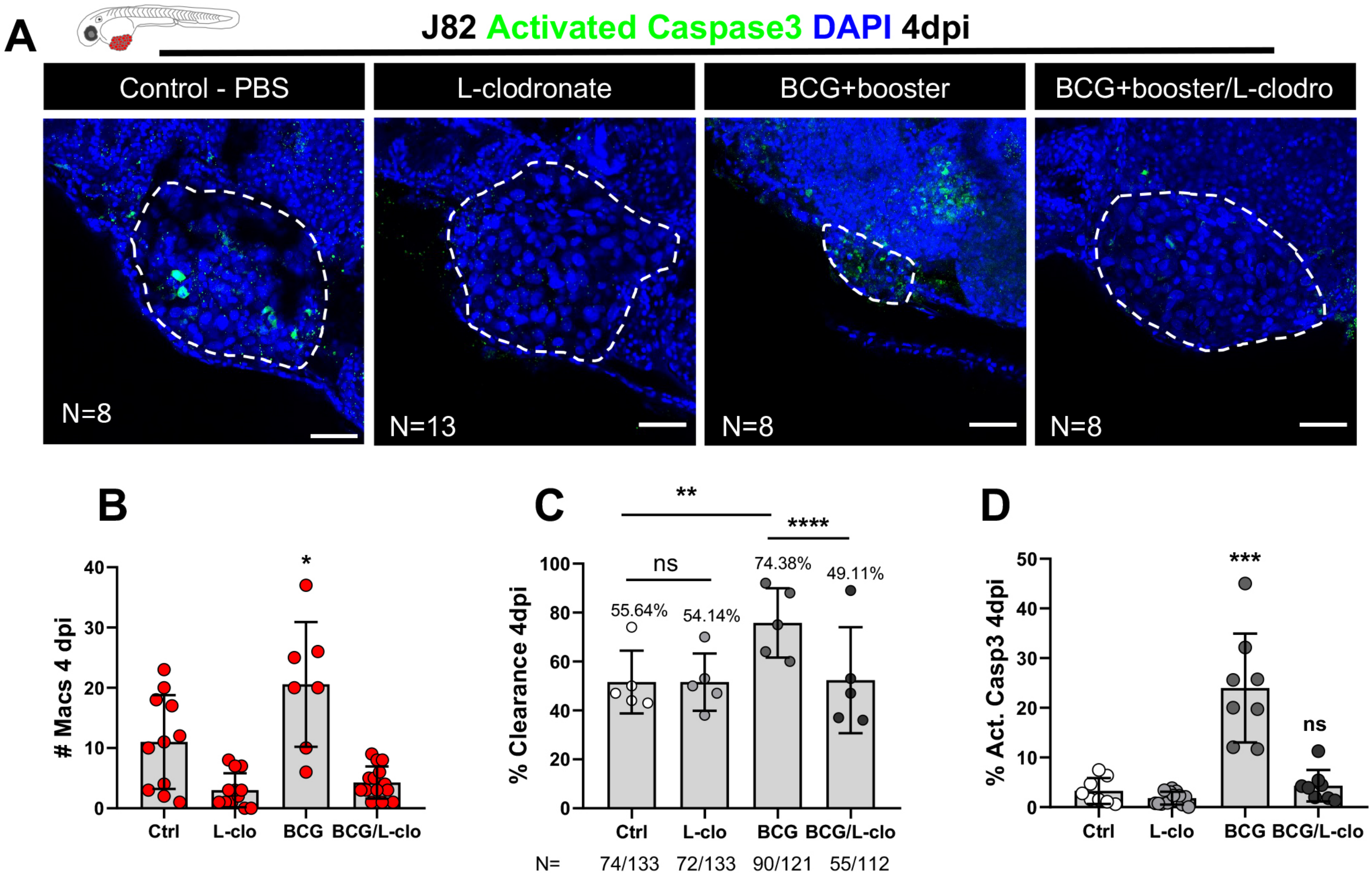

Fig. S4. Macrophages are essential for susceptibility to BCG immunotherapy of J82 zebrafish bladder cancer xenografts. A) Representative confocal images of MIBC-J82 xenografts, in which human cancer cells were labelled with the Deep Red Cell Tracker lipophilic staining (not shown) and were stained for the apoptosis marker activated caspase 3 (green) with DAPI nuclei counterstaining in BCG/L-clodronate experiments at 4dpi. **B)** Quantification of the absolute numbers of infiltrating macrophages in BCG/L-clodronate experiments (*P=0.0461). **C)** Quantification of the percentage of clearance in BCG/L-clodronate experiments at 4dpi (**P=0.0091, ****P<0.0001). Bars indicate the results as AVG ±STDEV and each dot represents a full round of injections in which N= # of xenografts without tumor at 4dpi/ total number of xenografts at 4dpi**. D)** Quantification of the percentage of apoptosis/activated caspase3 positive cells in BCG/ L-clodronate experiments at 4dpi (***P=0.0002). Bars indicate the results as AVG±STDEV and each dot represents one xenograft pooled from 3 independent experiments. Number of analyzed xenografts is indicated in the images. Clearance data set was analyzed using Fisher's exact test. Percentage of activated caspase 3 and macrophage numbers data sets with a Gaussian distribution were analyzed by parametric unpaired t-test and data sets that did not pass the normality tests were analyzed by nonparametric unpaired Mann–Whitney test. Unless stated otherwise, each experimental dataset was challenged to the respective control. Differences were considered significant at P < 0.05 and statistical output was represented as follows: non-significant (ns) ≥0.05, *<0.05, **<0.01, ***<0.001, ****<0.0001. Additionally, **B)** and **D)** were analyzed with Welch's ANOVA in which the P values were 0.0011 and 0.0006,respectively. All images are anterior to the left, posterior to right, dorsal up and ventral down. White dashes outline the tumor. Scale bar: 50 µm. dpi: days post-injection. Note: this experiment was performed in parallel with Figure 1, thus they share the same controls.

Disease Models & Mechanisms • Supplementary information

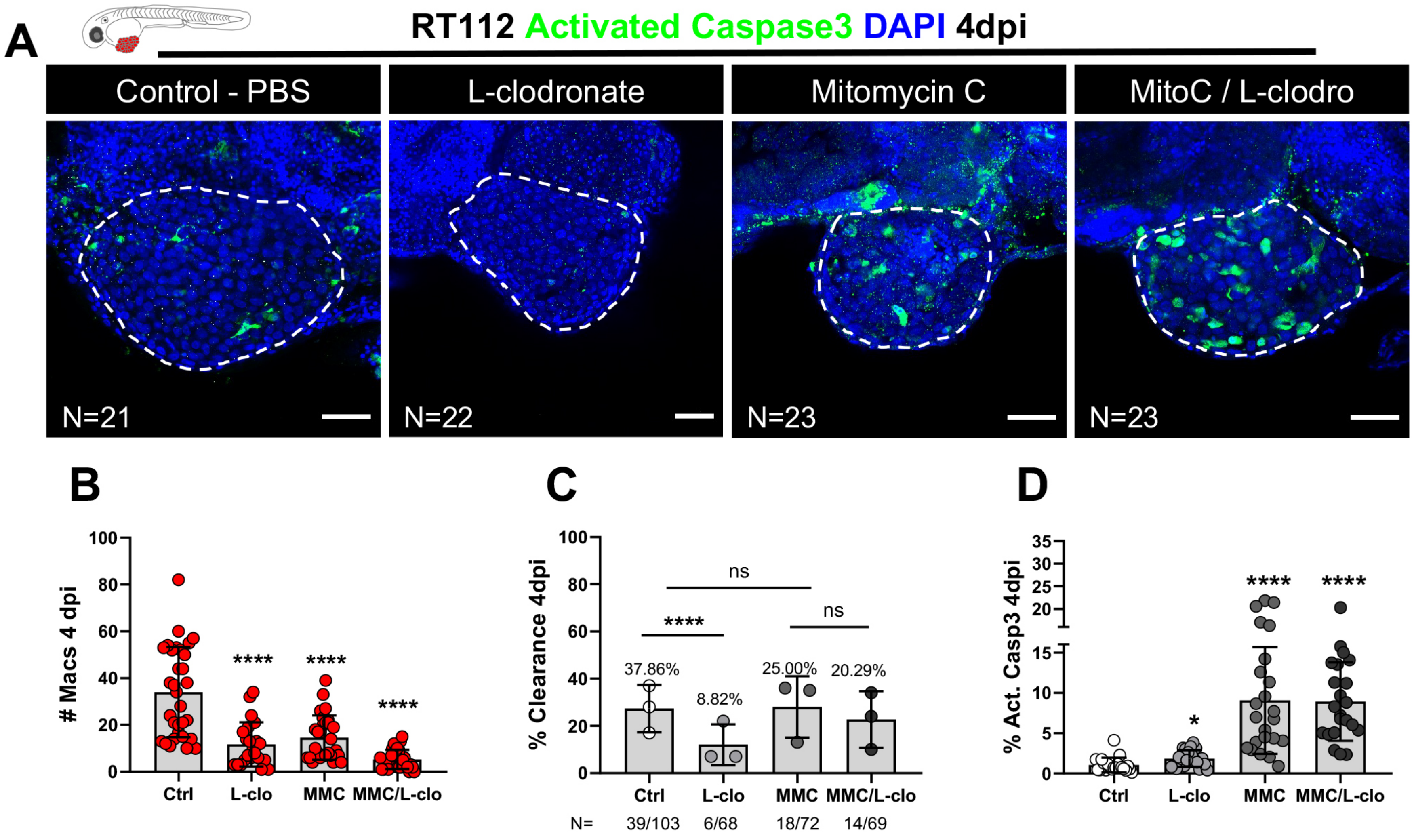

Fig. S5. Cytotoxic effects of Mitomycin C in zebrafish bladder cancer xenografts are not mediated by macrophages. A) Representative confocal images of NMIBC- RT112 xenografts, in which human cancer cells were labelled with the Deep Red Cell Tracker lipophilic staining (not shown) and were stained for the apoptosis marker activated caspase 3 (green) and DAPI nuclei counterstaining in MMC/L-clodronate experiments at 4dpi. **B)** Quantification of the absolute numbers of infiltrating macrophages in MMC/L-clodronate experiments (****P<0.0001). **C)** Quantification of the percentage of clearance in MMC/L-clodronate experiments at 4dpi (****P<0.0001). Bars indicate the results as AVG±STDEV and each dot represents a full round of injections, in which N= # of xenografts without tumor at 4dpi/ total number of xenografts at 4dpi**. D)** Quantification of the percentage of apoptosis/activated caspase3 positive cells in MMC/L-clodronate experiments at 4dpi (*P=0.0127, ****P<0.0001). Bars indicate the results as AVG±STDEV and each dot represents one xenograft pooled from 3 independent experiments. Number of analyzed xenografts is indicated in the images. Clearance data set was analyzed using Fisher's exact test. Percentage of activated caspase 3 and macrophage numbers data sets with a Gaussian distribution were analyzed by parametric unpaired t-test and data sets that did not pass the normality tests were analyzed by nonparametric unpaired Mann–Whitney test. Unless stated otherwise, each experimental dataset was challenged to the respective control. Differences were considered significant at $P < 0.05$ and statistical output was represented as follows: non-significant (ns) ≥0.05, *<0.05, **<0.01, ***<0.001, ****<0.0001. Additionally, **B)** and **D)** were analyzed with Welch's ANOVA in which the P values were <0.0001 for both the percentage of apoptosis and the number of infiltrating macrophages. White dashes outline the tumor. All images are anterior to the left, posterior to right, dorsal up and ventral down. Scale bar: 50 µm. dpi: days post-injection. MMC: Mitomycin C. Note: these experiments were performed in parallel with Figure 3, thus they share the same controls.

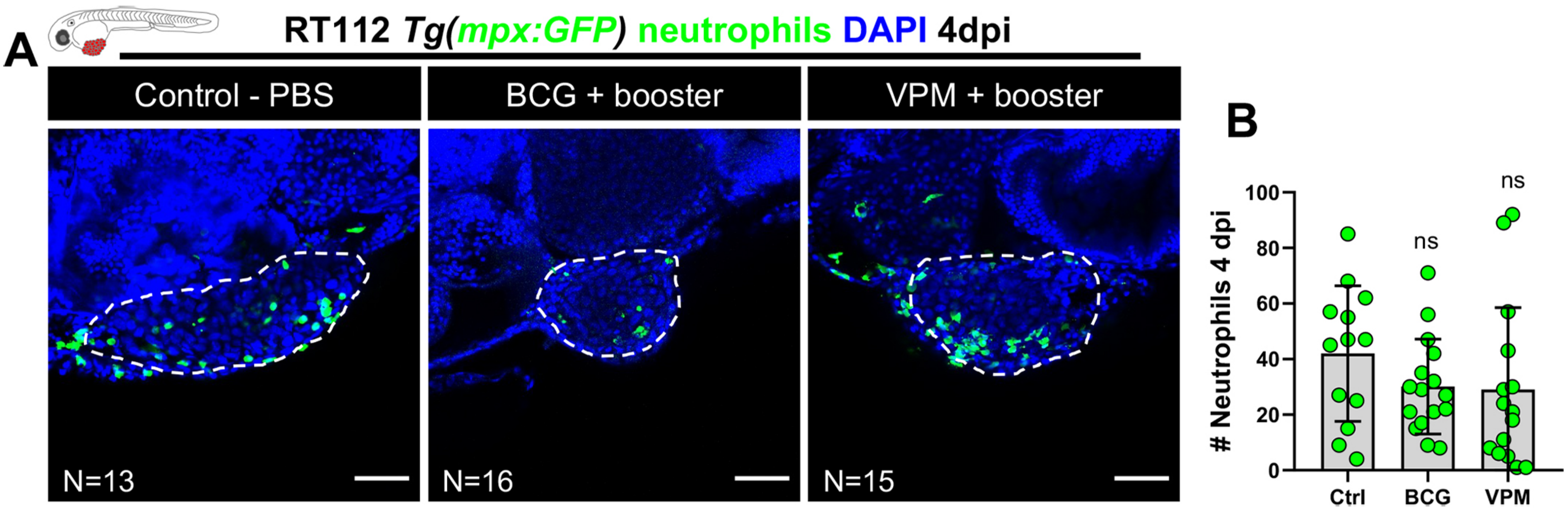

Fig. S6. BCG treatment has no significant effects in neutrophil infiltration at 4dpi. A) Representative confocal images of neutrophils (green) in NMIBC-RT112 control and BCG+booster- or VPM1002+booster-treated xenografts, in which human cancer cells were labelled with the Deep Red Cell Tracker lipophilic staining (not shown) at 4dpi. **B)** Quantification of the absolute numbers of infiltrating neutrophils at 4dpi. Bars indicate the results as AVG±STDEV and each dot represents one xenograft pooled from 2 independent experiments. Number of analyzed xenografts is indicated in the images. Neutrophil numbers data set with a Gaussian distribution was analyzed by parametric unpaired t-test and data set that did not pass the normality tests was analyzed by nonparametric unpaired Mann–Whitney test. Unless stated otherwise, each experimental dataset was challenged to the respective control. Differences were considered significant at $P < 0.05$ and statistical output was represented as follows: non-significant (ns) ≥0.05, *<0.05, **<0.01, ***<0.001, ****<0.0001. Additionally, **B)** was analyzed with Welch's ANOVA for which the P value was 0.3153. White dashes outline the tumor. All images are anterior to the left, posterior to right, dorsal up and ventral down. Scale bar: 50 µm. dpi: days post-injection. Note: the quantifications presented in this figure are also represented in Fig. 7E.

Disease Models & Mechanisms • Supplementary information

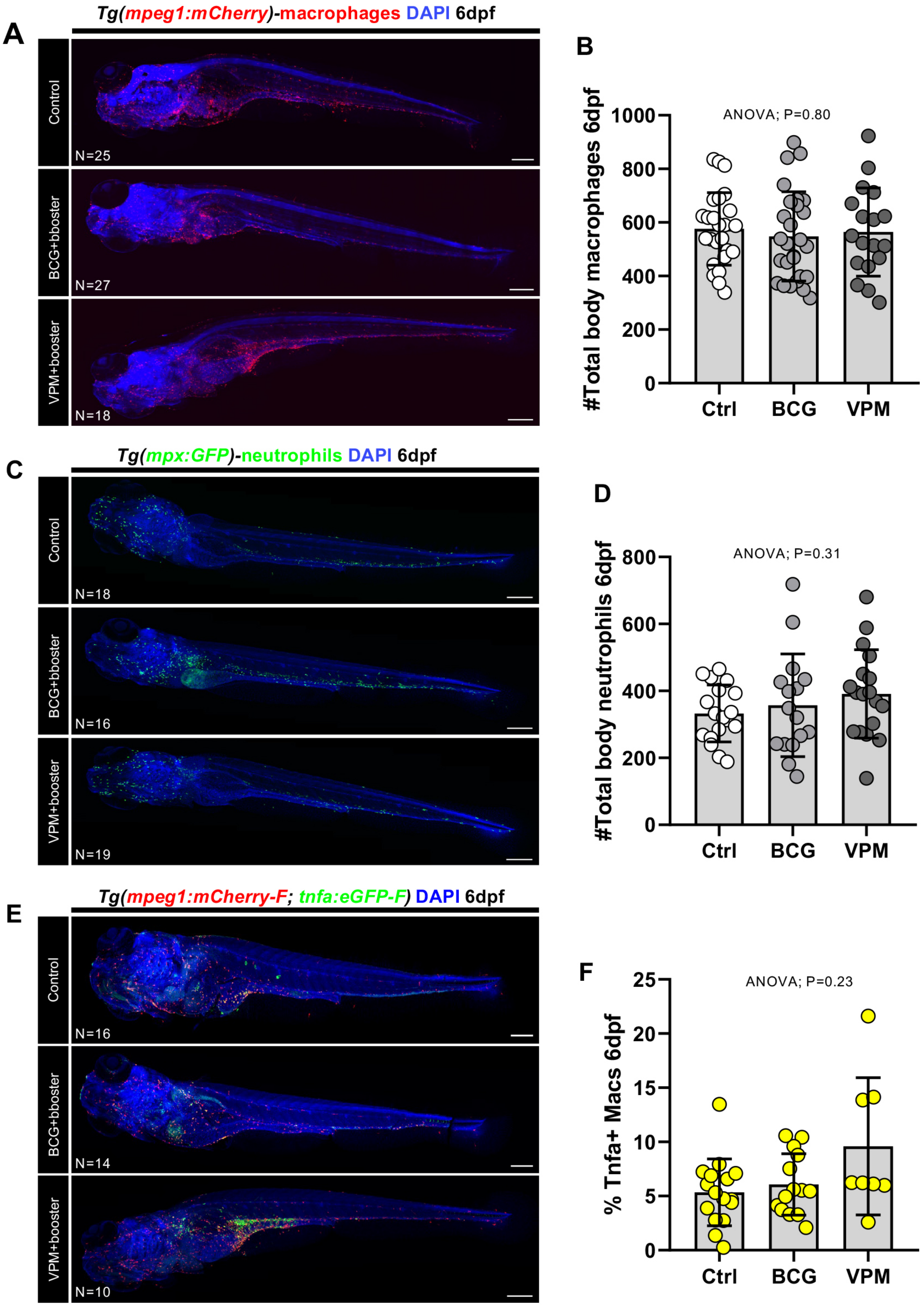

Disease Models & Mechanisms • Supplementary information

Fig. S7. BCG treatment has no significant effects in neutrophil nor macrophage distribution and polarization in zebrafish larvae. Representative full body confocal images of macrophages (**A**, red), neutrophils (**C**, green), and double transgenics for macrophages (red) and Tnfa expression (green) (**E**) of control and BCG+booster- or VPM1002 +booster- treated larvae at 6dpf. Quantification of the absolute number of total body macrophages (**B,** Welch's ANOVA P=0.80) and neutrophils (**D,** Welch's ANOVA P=0.31). Quantification of the percentage of Tnfa positive macrophages in the larvae's body (**F**, Welch's ANOVA P=0.23). Bars indicate the results as AVG±STDEV and each dot represents one larvae pooled from 2 independent experiments. All images are anterior to the left, posterior to right, dorsal up and ventral down. Scale bar: 200µm. dpf: days post-fertilization.

Disease Models & Mechanisms • Supplementary information

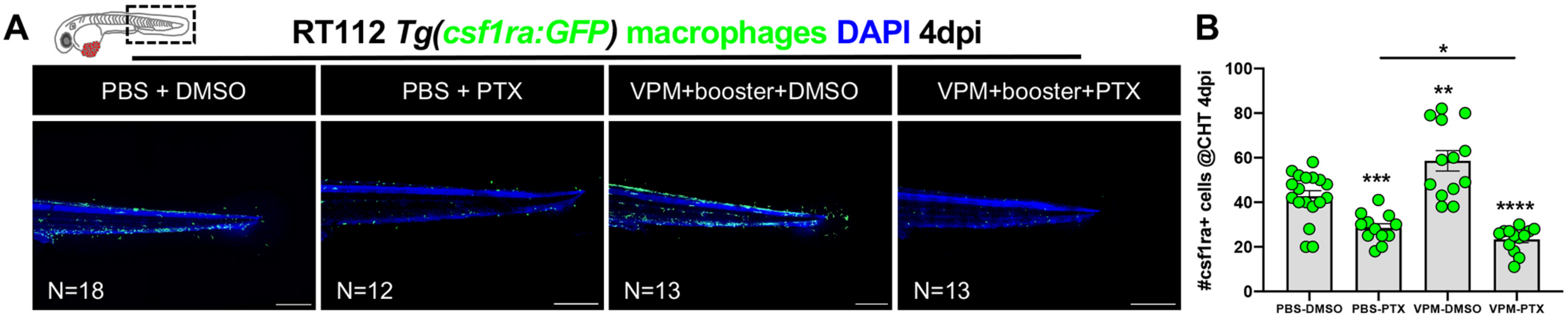

Fig. S8. Tnf inhibition abrogates the induction of in csf1ra+ cells by VPM1002 injection. A) Representative confocal images of macrophages (green) in the CHT of NMIBC-RT112 control and VPM1002+booster-treated xenografts exposed to either DMSO or PTX at 4 dpi. **B)** Quantification of the absolute numbers of csf1ra+ macrophages in the CHT of NMIBC-RT112 control and VPM1002+booster-treated xenografts exposed to either DMSO or PTX at 4 dpi (*P=0.0466, **P=0.0064, ***P=0.0001, ****P<0.0001). Bars indicate the results as AVG±STDEV and each dot represents one xenograft pooled from 2 independent experiments. Data sets with a Gaussian distribution were analyzed by parametric unpaired t-test and data sets that did not pass the normality tests were analyzed by nonparametric unpaired Mann–Whitney test. Unless stated otherwise, each experimental dataset was challenged to the respective control. Additionally, **B)** was analyzed with Welch's ANOVA for which the P value was <0.0001. All images are anterior to the left, posterior to right, dorsal up and ventral down. Scale bar: 250 µm. dpi: days post-injection. CHT: caudal hematopoietic tissue. DMSO: dimethyl sulfoxide. PTX: pentoxifylline.

Disease Models & Mechanisms • Supplementary information

Table S1. List of zebrafish lines used in each Figure.

Figures	Zebrafish Lines
Figure 1	*mitfa*^b692 *(nacre)*<br>*Tg(csf1ra:GFP)*<br>*Tg(mpx:GFP)*
Figure 2	*Tg(mpeg1:mCherry)*<br>*Tg(mpx:GFP)*<br>*Tg(csf1ra:GFP)*<br>*Tg (mpeg1:mCherry-F; tnfa:eGFP-F)*
Figure 3	*Tg(mpeg1:mCherry)*<br>*Tg(csf1ra:GFP)*<br>*Tg(mpx:GFP)*<br>*Tg(fli:GFP)*
Figure 4	*Tg(mpeg1:mCherry)*<br>*Tg(csf1ra:GFP)*<br>*Tg(mpx:GFP)*<br>*mitfa*^b692 *(nacre)*<br>*Tg(mpeg1:mCherry-F; tnfa:eGFP-F)*
Figure 5	*Tg(csf1ra:GFP)*
Figure 6	*Tg(mpeg1:mCherry)*<br>*Tg(csf1ra:GFP)*<br>*Tg(mpx:GFP)*
Figure 7	*Tg(mpeg1:mCherry)*<br>*Tg(mpx:GFP)*<br>*Tg(csf1ra:GFP)*<br>*Tg(mpeg1:mCherry; mpx:GFP)*
Figure 8	*Tg(csf1ra:GFP)*<br>*Tg(mpeg1:mCherry; nfkb:GFP)*<br>*Tg(mpeg1:mCherry-F; tnfa:eGFP-F)*
Supplementary Figure 1	*Tg(csf1ra:GFP)*<br>*Tg(mpx:GFP)*
Supplementary Figure 3	*Tg(mpeg1:mCherry)*<br>*Tg(csf1ra:GFP)*<br>*Tg (mpeg1:mCherry-F; tnfa:eGFP-F)*
Supplementary Figure 4	*mitfa*^b692 *(nacre)*<br>*Tg(mpx:GFP)*<br>*Tg(csf1ra:GFP)*<br>*Tg(mpeg1:mCherry; nfkb:GFP)*
Supplementary Figure 5	*Tg(csf1ra:GFP)*<br>*Tg(mpeg1:mCherry; nfkb:GFP)*<br>*Tg(mpx:GFP)*
Supplementary Figure 6	*Tg(mpx:GFP)*
Supplementary Figure 7	*Tg(mpx:GFP)*<br>*Tg(mpeg1:mCherry)*<br>*Tg(csf1ra:GFP)*<br>*Tg (mpeg1:mCherry-F; tnfa:eGFP-F)*
Supplementary Figure 8	*Tg(csf1ra:GFP)*

Disease Models & Mechanisms • Supplementary information

Table S2. List of human and zebrafish primers used for RT-qPCR.

Organism	Gene	NCBI Gene ID	Primer	Nucleotide sequence (5'→3')
Human	*TNFα*	7124	Forward 1	CTCTTCTGCCTGCTGCACTTTG
			Reverse 1	ATGGGCTACAGGCTTGTCACTC
			Forward 2	CCCCAGGGACCTCTCTCTAATC
			Reverse 2	GGTTTGCTACAACATGGGCTACA
	LTα	4049	Forward 1	CTCCTGCACCTGCTGCCTGGATC
			Reverse 1	GAAGAGACGTTCAGGTGGTGTCAT
			Forward 2	CATCTACTTCGTCTACTCCCAGG
			Reverse 2	CCCCGTGGTACATCGAGTG
	TNFRSF1A	7132	Forward	AACGAGTGTGTCTCCTGTAGT
			Reverse	GGAGTAGAGCTTGGACTTCCAC
	TNFRSF1B	7133	Forward 1	TGAAACATCAGACGTGGTGTG
			Reverse 1	TGCAAATATCCGTGGATGAAGTC
			Forward 2	TTCATCCACGGATATTTGCAGG
			Reverse 2	GCTGGGGTAAGTGTACTGCC
	TNFRSF21	27242	Forward	TTGACTGACCGAGAATGCACT
			Reverse	TTCATCACACTAGAAGGCACATC
	C1QTNF6	114904	Forward	TGCCTGAGATCAGACCCTACA
			Reverse	GCCCACTGAGAAGGCGAAG
	EEF1A1	1915	Forward	ATCCACCTTTGGGTCGCTTT
			Reverse	CAGCCTTCTTGTCCACTGCT

Disease Models & Mechanisms • Supplementary information

Organism	Gene	NCBI Gene ID	Primer	Nucleotide sequence (5'→3')
Zebrafish	*runx1*	58126	Forward	GTCACAGTGATGGCGGGAAA
			Reverse	GGTTCTTGATGGCGGCTGTA
	lmo2	30332	Forward	GATGCTTGGAATCTGGCGTACA
			Reverse	CCATCTGCCGCACAAAACG
	spi1b	30117	Forward	CAGAGCTACAAAGCGTGCAG
			Reverse	GCAGAAGGTCAAGCAGGAAC
	lcp1	30583	Forward	GCAGTGGGTGAACGAAACAC
			Reverse	CAGCAGGTCGTAGCGGATAG
	mpx	337514	Forward	GGGGCAGAAGAAGAAAGTC
			Reverse	TTTGCGCACCCTTGCTAAAC
	mpeg1.1	335407	Forward	GTGAAAGAGGGTTCTGTTACA
			Reverse	GCCGTAATCAAGTACGAGTT
	tnfa	405785	Forward	GCGCTTTTCTGAATCCTACG
			Reverse	TGCCCAGTCTGTCTCCTTCT
	il1b	405770	Forward	TGGACTTCGCAGCACAAAATG
			Reverse	GTTCACTTCACGCTCTTGGATG
	il6	100885851	Forward	CCTCTCCTCAAACCTTCAGACC
			Reverse	TGCTGTGTTTGATGTCGTTCAC
	ifng1	405790	Forward 1	ATGCAGAATGACAGCGTGGA
			Reverse 1	TTCCTTGATCGCCCATAGCG
			Forward 2	ATGATTGCGCAACACATGAT
			Reverse 2	ATCTTTCAGGATTCGCAGGA
	il10	553957	Forward	CCACAACCCCAATCGACTCC
			Reverse	AGCAAATCAAGCTCCCCCATA
	tgfb1b	359834	Forward	GCAGAAAACGGGAAACAGATGCT
			Reverse	ACAGACTTCTAACACAGCAACCCT
	eef1a1a	336334	Forward	TTCTGTTACCTGGCAAAGGG
			Reverse	TTCAGTTTGTCCAACACCCA

Disease Models & Mechanisms • Supplementary information

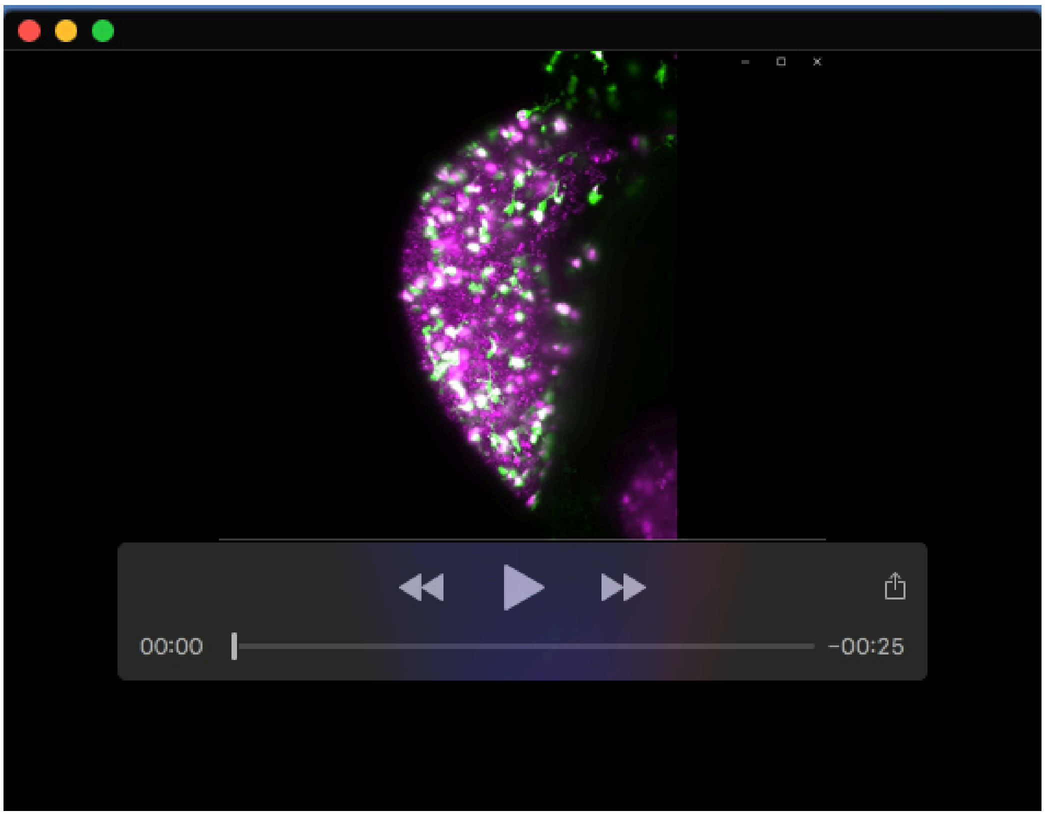

Movie 1. Macrophage kinetics of control NMIBC-RT112 zebrafish xenografts 1dpi. Maximum intensity projection of the tumor. Each colored line represents the path a single macrophage followed in a 15-hour time lapse. Images of the tumor were acquired in stacks of 5µm in the Z plain every 3 minutes. Tracking was made using the MaMut plugin from ImageJ/Fiji.

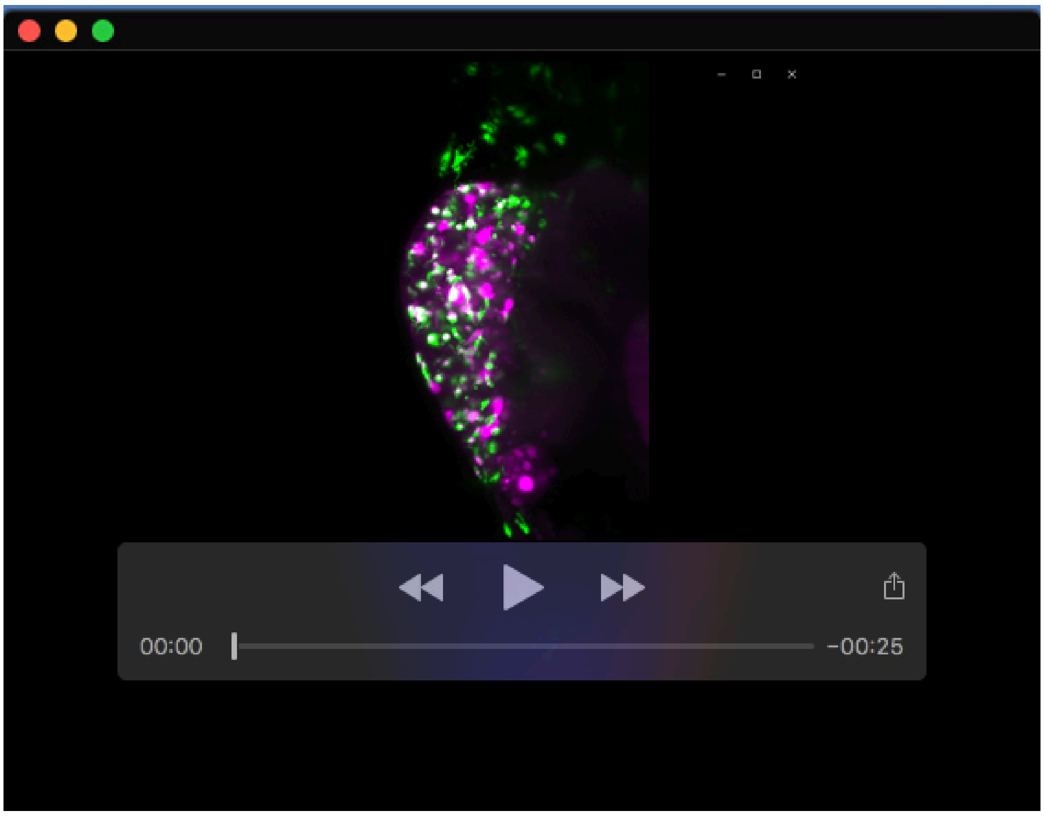

Movie 2. Macrophage kinetics of BCG treated NMIBC-RT112 zebrafish xenografts 1dpi. Maximum intensity projection of the tumor. Each colored line represents the path a single macrophage followed in a 15-hour time lapse. Images of the tumor were acquired in stacks of 5µm in the Z plain every 3 minutes. Tracking was made using the MaMut plugin from ImageJ/Fiji.

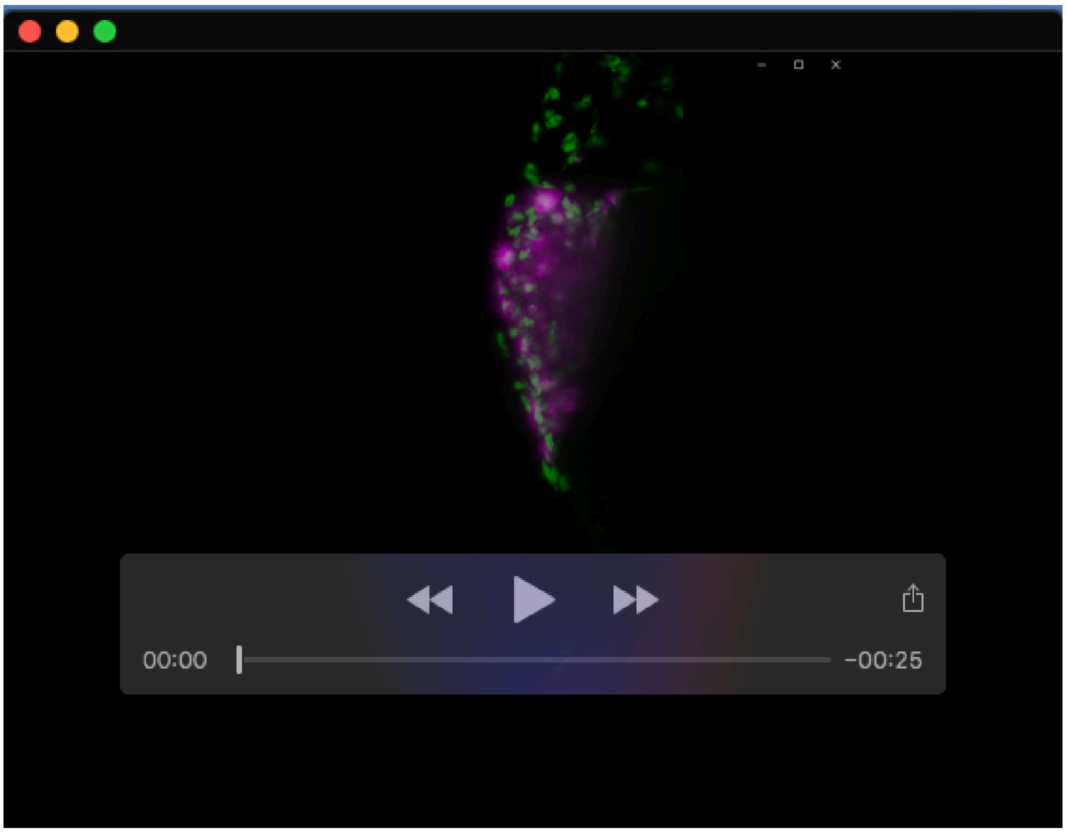

Movie 3. Macrophage kinetics of VPM1002 treated NMIBC-RT112 zebrafish xenografts 1dpi. Maximum intensity projection of the tumor. Each colored line represents the path a single macrophage followed in a 15-hour time lapse. Images of the tumor were acquired in stacks of 5µm in the Z plain every 3 minutes. Tracking was made using the MaMut plugin from ImageJ/Fiji.

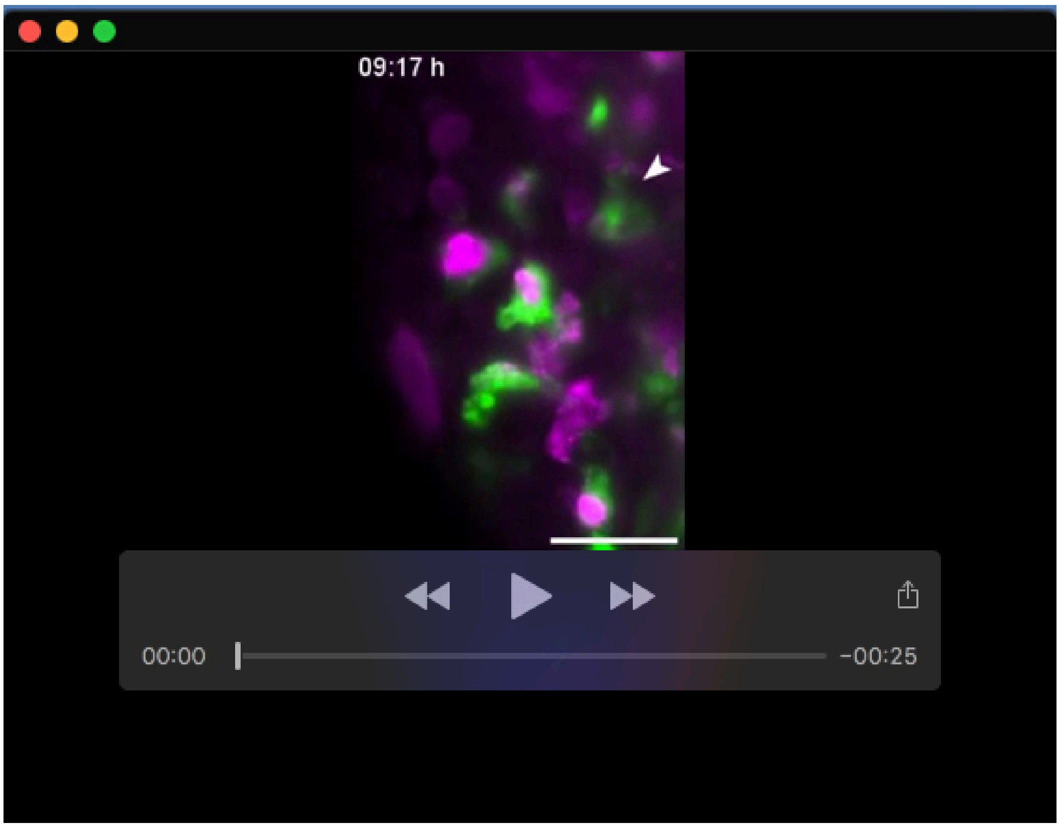

Movie 4. Macrophage touching in the TME of NMIBC-RT112 zebrafish xenografts. Representative video showing macrophages (labelled in green) phagocyting cancer cells (labelled in magenta) and actively touching their cell membranes within the tumor microenvironment of a 1dpi NMIBC-RT112 zebrafish xenograft.

Disease Models & Mechanisms • Supplementary information

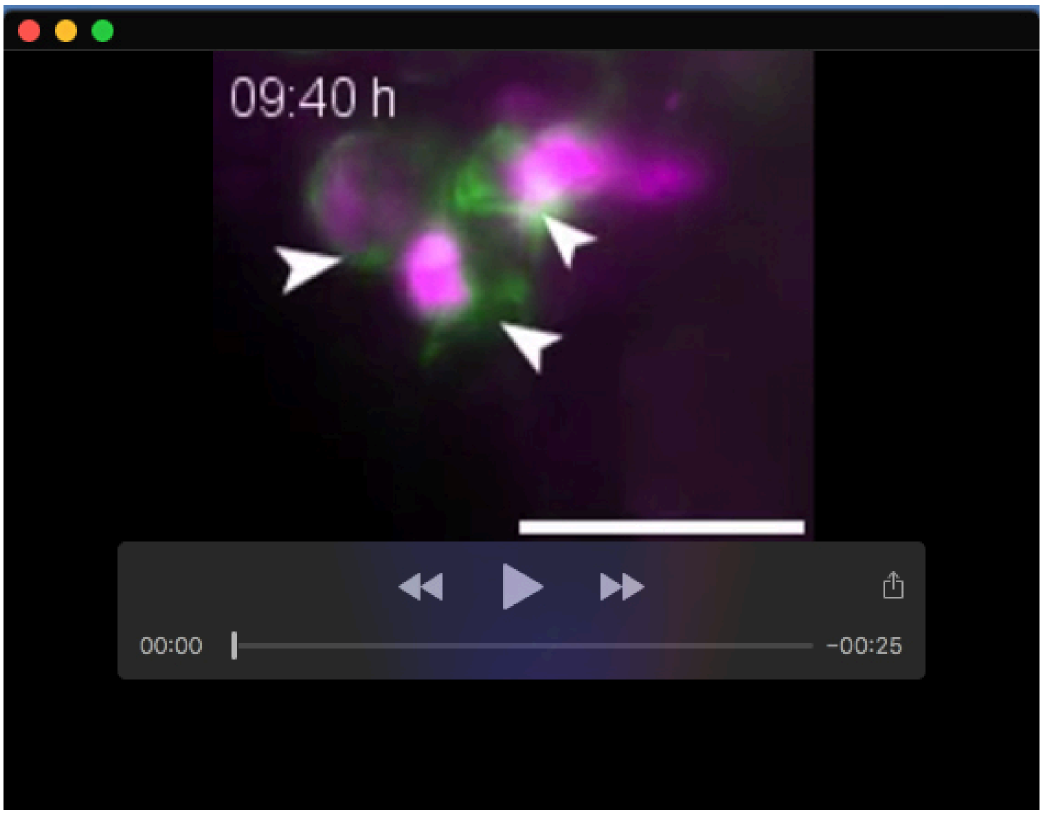

Movie 5. Macrophage fusion-like events in the TME of NMIBC-RT112 zebrafish xenografts. Representative video showing macrophages (labelled in green) phagocyting cancer cells (labelled in magenta) and joining their cell membranes within the tumor microenvironment of a 1dpi NMIBC-RT112 zebrafish xenograft.

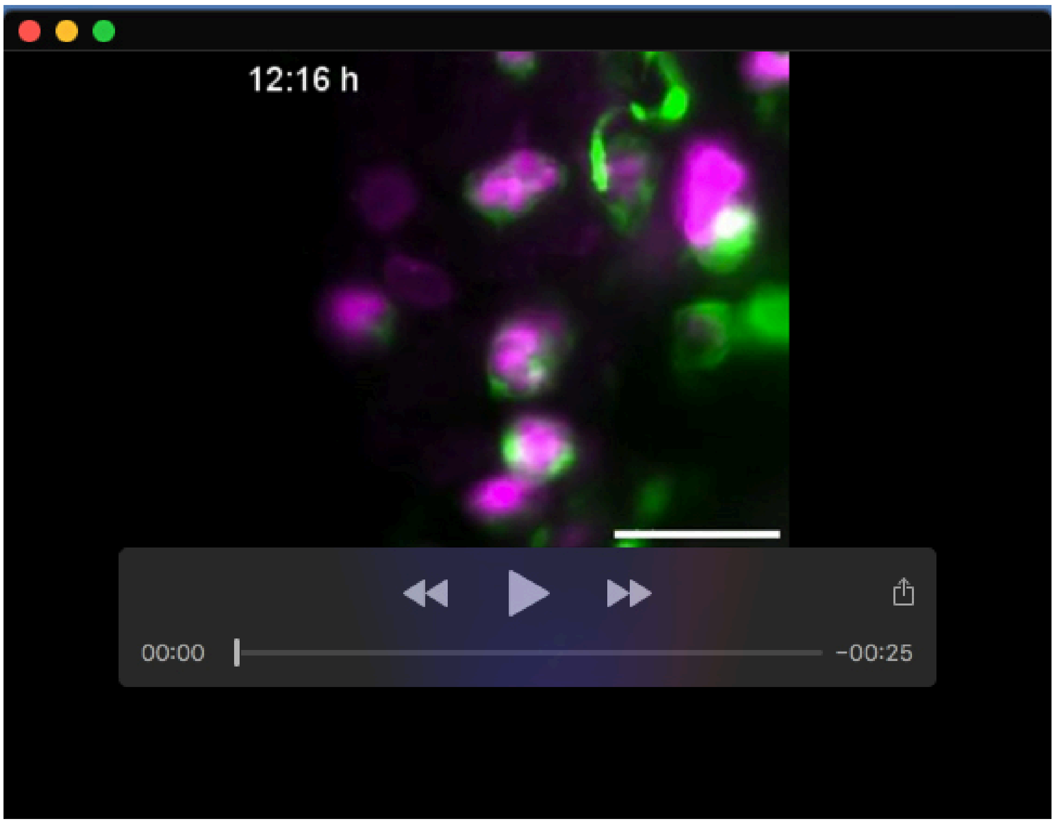

Movie 6. Dendritic-like cells in the TME of NMIBC-RT112 zebrafish xenografts. Representative video showing macrophages (labelled in green) and cancer cells (labelled in magenta) within the tumor microenvironment of a 1dpi bladder cancer xenograft. Dendritic-like cells with no phagocytic behavior can be seen actively interacting with their surrounding macrophages.

Disease Models & Mechanisms • Supplementary information
